# Essential Oils of *Zingiber* Species from Vietnam: Chemical Compositions and Biological Activities

**DOI:** 10.3390/plants9101269

**Published:** 2020-09-26

**Authors:** Le Thi Huong, Nguyen Thanh Chung, Trinh Thi Huong, Ly Ngoc Sam, Nguyen Huy Hung, Isiaka Ajani Ogunwande, Do Ngoc Dai, Le Duy Linh, William N. Setzer

**Affiliations:** 1 School of Natural Science Education, Vinh University, 182 Le Duan, Vinh City 43000, Nghệ An Province, Vietnam; leduylinhdhv@gmail.com; 2 Vietnam Academy of Science and Technology, Graduate University of Science and Technology, 18-Hoang Quoc Viet, Cau Giay, Hanoi 10072, Vietnam; chungpuhoat@gmail.com (N.T.C.); trinhthihuongtn@hdu.edu.vn (T.T.H.); lysamitb@gmail.com (L.N.S.); daidn23@gmail.com (D.N.D.); 3 Faculty of Natural Science, Hong Duc University, Thanh Hoa City 4000, Thanh Hoa Province, Vietnam; 4 Institute of Tropical Biology, Vietnam Academy of Science and Technology, 85-Tran Quoc Toan, 3 District, Ho Chi Minh City 7000, Vietnam; 5 Center for Advanced Chemistry, Institute of Research and Development, Duy Tan University, 03 Quang Trung, Da Nang 550000, Vietnam; nguyenhuyhung@duytan.edu.vn; 6 Foresight Institute of Research and Translation, University Road, Aleku Area, Osogbo 230271, Nigeria; isiakaogunwande@gmail.com; 7 Faculty of Agriculture, Forestry and Fishery, Nghe An College of Economics, 51-Ly Tu Trong, Vinh City 43000, Vietnam; 8 Aromatic Plant Research Center, 230 N 1200 E, Suite 100, Lehi, UT 84043, USA; 9 Department of Chemistry, University of Alabama in Huntsville, Huntsville, AL 35899, USA

**Keywords:** ginger, Aedes aegypti, Aedes albopictus, Culex quinquefasciatus, antibacterial, antifungal

## Abstract

Mosquito-borne diseases are a large problem in Vietnam as elsewhere. Due to environmental concerns regarding the use of synthetic insecticides as well as developing insecticidal resistance, there is a need for environmentally-benign alternative mosquito control agents. In addition, resistance of pathogenic microorganisms to antibiotics is an increasing problem. As part of a program to identify essential oils as alternative larvicidal and antimicrobial agents, the leaf, stem, and rhizome essential oils of several *Zingiber* species, obtained from wild-growing specimens in northern Vietnam, were acquired by hydrodistillation and investigated using gas chromatography. The mosquito larvicidal activities of the essential oils were assessed against *Culex quinquefasciatus*, *Aedes albopictus*, and *Ae. aegypti*, and for antibacterial activity against a selection of Gram-positive and Gram-negative bacteria, and for activity against *Candida albicans*. *Zingiber* essential oils rich in α-pinene and β-pinene showed the best larvicidal activity. *Zingiber nudicarpum* rhizome essential oil showed excellent antibacterial activity against *Enterococcus faecalis*, *Staphylococcus aureus*, and *Bacillus cereus*, with minimum inhibitory concentrations (MIC) of 2, 8, and 1 μg/mL, respectively. However, the major components, α-pinene and β-pinene, cannot explain the antibacterial activities obtained.

## 1. Introduction

Vietnam is located in the tropics of Southeast Asia, and several mosquito-borne diseases are endemic, including Japanese encephalitis [1], dengue fever [2], and Zika [3]. *Culex* species are considered to be important vectors of Japanese encephalitis virus (JEV), including *Culex quinquefasciatus* Say (Diptera: Culicidae) [4], but other mosquito genera may also serve as competent vectors of the virus [5,6]. Dengue fever is hyperendemic to Vietnam with all four serotypes of the virus in circulation, resulting in periodic acute epidemics of both dengue fever and dengue hemorrhagic fever [7,8]. *Aedes aegypti* (L.) and *Aedes albopictus* (Skuse) (Diptera: Culicidae) mosquitoes are the principal vectors of dengue fever virus (DFV) in Vietnam [9]. Zika virus (ZIKV) first appeared in Vietnam in 2016, where the primary transmission vector is *Aedes* mosquitoes [10]. Exacerbating this problem is the increasing insecticide resistance in *Aedes* [11,12,13] and *Culex* mosquitoes [14,15].

As observed throughout the world, antimicrobial resistance is an increasing problem in Vietnam [16]. Particularly noteworthy are antibiotic-resistant organisms in hospital settings, including *Klebsiella pneumoniae*, *Acinetobacter baumannii*, *Pseudomonas aeruginosa* [17], *Streptococcus pneumoniae*, *Haemophilus influenzae* [18], *Escherichia coli*, and *Staphylococcus aureus* [19].

*Zingiber* Mill. is a species-rich genus within the subfamily Zingireroideae of Zingiberaceae, which are native to Southeast Asia [20]. *The Plant List* currently has 146 accepted names for *Zingiber* species [21]. The phytochemistry, particularly essential oil chemistry, and the pharmacology of *Zingiber* have been reviewed [22]. Currently, at least thirty-six species of *Zingiber* have been reported in Vietnam [23,24,25].

There is a need to discover new and alternative insect-control agents and antimicrobial agents. In this work, essential oils from seven species of *Zingiber* growing in Vietnam were collected and analyzed by gas chromatographic methods. Six species were screened for mosquito larvicidal activity and antibacterial and antifungal activity.

*Zingiber cornubracteatum* Triboun & K. Larsen was first recorded in northern Thailand (Mae Hong Son) [26], but has since been collected in northern Vietnam (Thanh Hoa, Nghe An, and Quang Binh provinces) [25]. There have been no reports on the phytochemistry of this plant. *Zingiber neotruncatum* T.L. Wu, K. Larsen & Turland has been recorded from southern and western Yunnan province, China [27] and Nghệ An province, Vietnam [28]. There have been no previous reports on the essential oil of this species. *Zingiber nitens* M.F. Newman is known from Bolikhamsai Province, Laos [29], and Nghệ An Province, Vietnam [30]. The essential oil composition of *Z. nitens* from Vietnam has been previously published [31]. *Zingiber nudicarpum* D. Fang has been recorded in Guangxi Province, China [27], Laos [32], and Vietnam [33]. The essential oil composition of *Z. nudicarpum* from Vietnam has been previously published [34]. *Zingiber ottensii* Valeton is native to Southeast Asia, including Thailand, Indonesia, Malaysia, Laos, Myanmar, and Vietnam [35,36]. Essential oil compositions of *Z. ottensii* have been reported from Malaysia [37,38], Thailand [39,40], and Indonesia [41]. *Zingiber recurvatum* S.Q. Tong & Y.M. Xia has been recorded in southern Yunnan Province, China [42], northern Laos [32], and Vietnam [43,44]. There are apparently no reports on the volatile phytochemistry of this plant. *Zingiber vuquangensis* Lý N.S., Lê T.H., Trịnh T.H., Nguyễn V.H., Đỗ N.Đ. is a new species, only recently recorded in Vietnam [45]. The essential oil composition of *Z. vuquangensis* has been reported [46].

## 2. Results and Discussion

### 2.1. Essential Oil Compositions

**Figure 1:**
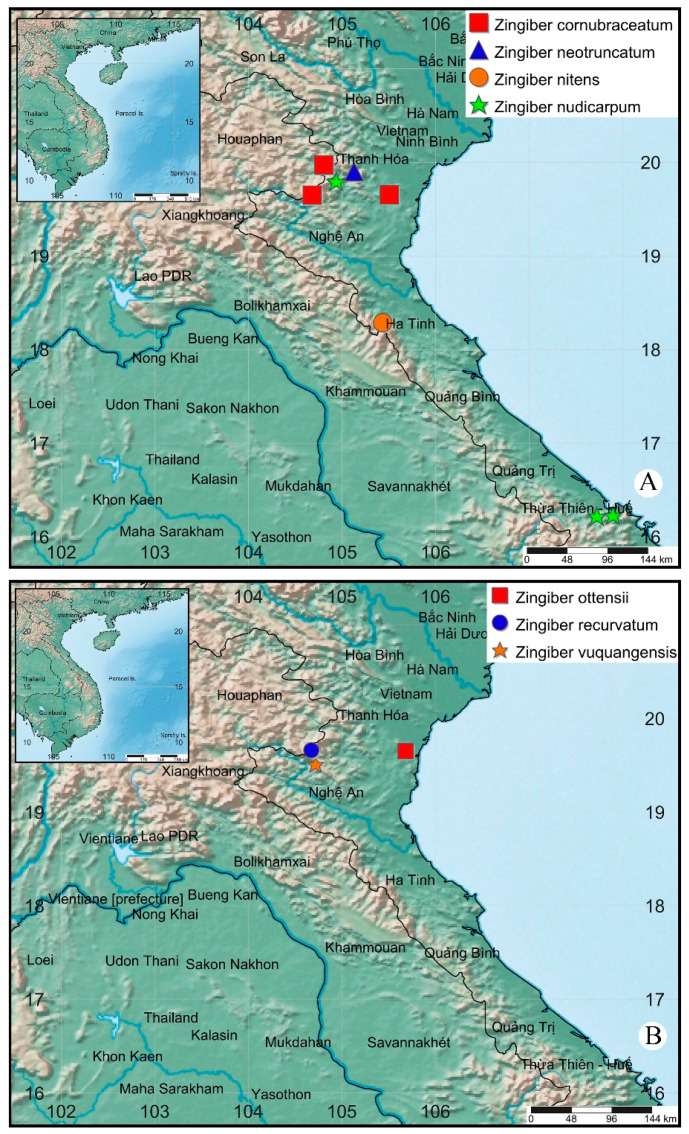
Distribution map of *Zingiber* species from the north-central Vietnam. (**A**): *Zingiber cornubraceatum* Triboun & K. Larsen (red squares); *Z. neotruncatum* Triboun & K. Larsen (blue triangle), *Z. nitens* M.F. Newman (orange circle), *Z. nudicarpum* D.Fang (green stars). (**B**): *Zingiber ottensii* Valeton (red square), *Z.* *recurvatum* S.Q. Tong & Y.M. Xia (blue circle), *Z.* *vuquangensis* Ly N.S., Le T.H., Trinh T.H., Nguyen V.H., Do N.D. (orange star).

The *Zingiber* plant tissues (leaves, stems, or rhizomes) were collected from north-central Vietnam and the plant tissues subjected to hydrodistillation to obtain the respective essential oils (Table 1, Figure 1). Gas chromatographic–mass spectral (GC-MS) and gas chromatographic–flame ionization detection (GC-FID) were used to analyze the essential oil compositions, which are compiled in Table 2.

The rhizome essential oils of *Z. cornubracteatum* were predominantly composed of monoterpene hydrocarbons and oxygenated monoterpenoids. The major components were α-pinene (8.2–14.5%), β-pinene (8.8–33.1%), limonene (1.0–5.1%), 1,8-cineole (2.5–10.4%), and linalool (0.4–31.0%). Both α-pinene and β-pinene were major components in the leaf essential oils of *Z. cornubracteatum* (2.7–10.1% and 18.8–67.3%, respectively). However, sesquiterpene hydrocarbons, including (*E*)-caryophyllene (1.8–13.9%), germacrene D (0.7–13.7%), bicyclogermacrene (2.7–18.9%), as well as the sesquiterpenoid (*E*)-nerolidol (0.9–23.0%), were also abundant in the leaf essential oils.

The leaf essential oils of *Z. nudicarpum* were also rich in α-pinene (5.0–10.9%), β-pinene (0.7–34.0%), in addition to the sesquiterpene hydrocarbons (*E*)-caryophyllene (6.4–24.3%), α-humulene (2.1–6.4%), germacrene D (0.6–6.5%), and bicyclogermacrene (3.3–16.1%). The leaf essential oil of *Z. nudicarpum* from Pù Hoạt Nature Reserve previously reported also showed α- and β-pinenes (2.4% and 11.7%, respectively) [34]. Important differences are apparent between the previously reported essential oil and those from the present study. The previous report found no (*E*)-caryophyllene or germacrene D, but large concentrations of cedrol (14.8%) and β-eudesmol (13.8%), which were not observed in this current study. Interestingly, the stem essential oils of *Z. nudicarpum* showed wide variation in monoterpene hydrocarbon concentrations, with the sample from Pù Hoạt Nature Reserve showing only low concentrations of monoterpene hydrocarbons compared to samples from Nam Đông or Bạch Mã National Park. For example, the concentrations were: α-pinene (0.0, 10.6, 6.1%), β-pinene (0.5, 9.0, 5.6%), *p*-cymene (0.0, 6.0, 0.1%), and limonene (0.0, 2.1, 6.0%). The Pù Hoạt stem essential oil had a high concentration of (*E*)-caryophyllene (52.6%). The rhizome essential oil from Pù Hoạt Nature Reserve had α-pinene (18.7%) and β-pinene (58.3%) as dominant constituents, and is qualitatively similar to a sample earlier reported from that collection site [34]. α-Pinene and β-pinene concentrations were lower in the rhizome essential oil sample from Nam Đông (4.0% and 9.8%, respectively).

The most abundant constituents in the rhizome essential oil of *Z. neotruncatum* were perillene (51.3%), neral (12.3%), and geranial (17.0%). Perillene is a major component of *Perilla frutescens* (perillene chemotype) [47] and *Elsholtzia polystachya* (perillene chemotype) [48], but has been found in *Zingiber* essential oils in small concentrations, e.g., *Z. officinale* rhizome oil (0.1–0.6%) [40,49,50] and *Z. zerumbet* leaf oil (1.2%) [51]. Neral and geranial are also major components of *Z. officinale* rhizome oil [49,50].

Camphene (40.4%) dominated the rhizome essential oil composition of *Z. nitens*, followed by bornyl acetate (14.5%), (*E*)-β-ocimene (12.7%), and α-pinene (10.5%). In comparison, the rhizome essential oil of *Z. nitens* from Pù Mát National Park previously reported contained bornyl acetate (11.8%), (*E*)-β-ocimene (1.1%), and α-pinene (7.3%), along with β-pinene (21.0%), and δ-elemene (12.8%) [31]. In contrast, the leaf essential oil of *Z. nitens* was composed largely of α-zingiberene (17.4%), α-pinene (11.2%), β-sesquiphellandrene (10.1%), (*E*)-nerolidol (10.0%), zingiberenol (7.2%), β-pinene (6.0%), and *ar*-curcumene (5.2%). The previously reported *Z. nitens* leaf essential oil was devoid of α-zingiberene, β-sesquiphellandrene, zingiberenol, and *ar*-curcumene, but contained large concentrations of δ-elemene (17.0%) and ledol (8.1%), which were not observed in the present sample. In addition, concentrations of *trans*-β-elemene, germacrene D, and bicyclogermacrene were high in the previous report (8.8%, 8.2%, and 8.3%, respectively), but low in the present sample (0.8%, 0.7%, and 1.5%, respectively). The variations in chemical constituents can likely be attributed to the different geographical collection sites as well as climatic factors. The Pù Mát sample was collected in May, 2014 (beginning of the rainy season), while the sample from Vũ Quang National Park (this work) was collected in September, 2018 (height of the rainy season).

**Figure 2:**
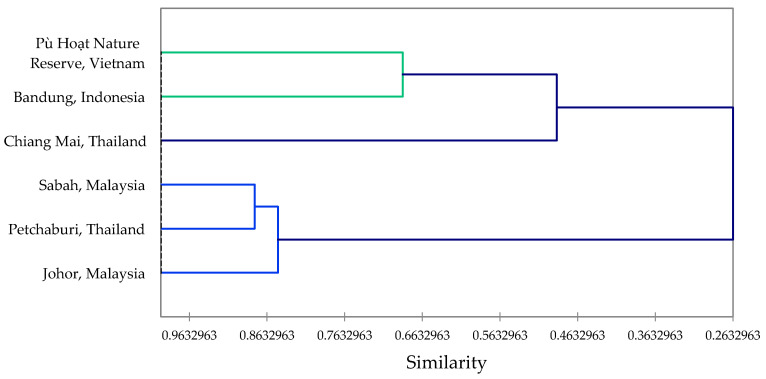
Dendrogram obtained from agglomerative hierarchical cluster analysis of the rhizome essential oils from *Zingiber ottensii* from different geographical locations.

The essential oil from the leaves of *Z. ottensii* had abundant sesquiterpene hydrocarbons, including (*E*)-caryophyllene (28.0%) and *trans*-β-elemene (17.0%), along with the monoterpene β-pinene (17.1%). (*E*)-Caryophyllene (19.6%) and *trans*-β-elemene (12.3%) were also found to be key constituents in *Z. ottensii* leaf oil from Bandung, West Java, Indonesia [41]. However, zerumbone was a major component (11.4%) in the leaf oil from Indonesia, but was not detected in the sample from Vietnam. The rhizome essential oil from Vietnam had monoterpenes as the major components, such as sabinene (21.6%), β-pinene (11.7%), γ-terpinene (5.5%), and terpinen-4-ol (17.1%), in addition to the sesquiterpenoid zerumbone (12.5%). Rhizome essential oils of *Z. ottensii* from other geographical locations have been reported; the major components are presented in Table 3. A hierarchical cluster analysis using the eight major constituents (Figure 2) reveals the similarities between these rhizome essential oils. The clusters are largely defined by the zerumbone concentrations.

The monoterpenes α-pinene (16.3%) and β-pinene (71.6%) dominated the leaf essential oil composition of *Z. recurvatum*. The major components in the rhizome essential oil of *Z. recurvatum* were (*E*)-caryophyllene (11.3%), bornyl acetate (10.4%), α-humulene (6.9%), and bicyclogermacrene (5.1%).

*Zingiber vuquangensis* leaf and rhizome essential oils were both rich in α-pinene (11.3% and 9.8%, respectively) and β-pinene (38.5% and 45.0%, respectively). The sesquiterpene hydrocarbons *trans*-β-elemene (5.9% and 10.0%), and (*E*)-caryophyllene (12.2% and 14.4%) were major components in the leaf and stem essential oils, respectively. The leaf, stem, and rhizome essential oils from *Z. vuquangensis* from Vu Quang National Park, Ha Tinh Province, Vietnam, have been previously published [46]. A comparison of the major components is summarized in Table 4. Although there are qualitative similarities in the essential oil compositions from these two collections (α-pinene, β-pinene, and (*E*)-caryophyllene are major components), there are some notable differences. Bornyl acetate and zerumbone were major components in the rhizome essential oil from the Vu Quang collection, but were not observed in the Pù Hoạt sample; *trans*-β-elemene was observed in relatively small concentrations in the sample from Vu Quang, but was a major component in the leaf and stem essential oils from Pù Hoạt. The differences in chemical composition can be attributed to the geographical locations of the two collections and/or the season when the samples were collected. The Vu Quang sample was collected in August, 2014 (rainy season), while the Pù Hoạt sample was collected in April, 2019 (dry season).

### 2.2. Mosquito Larvicidal Activity

Several of the *Zingiber* essential oils (depending on availability) were assayed for insecticidal activity against larvae of *Aedes aegypti*, *Aedes albopictus*, and *Culex quinquefasciatus* mosquitoes. The 24- and 48-h larvicidal activities are presented in Table 5.

The essential oils showing the best larvicidal activity against *Ae. aegypti* were *Z. cornubracteatum* rhizome essential oil from Bến En National Park (24-h LC_50_ = 17.0 μg/mL) and *Z. nudicarpum* leaf essential oil from Pù Hoạt Nature Reserve (24-h LC_50_ = 19.3 μg/mL). The rhizome essential oil of *Z. cornubracteatum* also demonstrated remarkable activity against *Ae. albopictus* (24-h LC_50_ = 12.7 μg/mL). Both *Z. nudicarpum* leaf essential oil and rhizome essential oil were very active against *Cx. quinquefasciatus* larvae, with LC_50_ values of 12.4 and 11.5 μg/mL, respectively.

**Figure 3:**
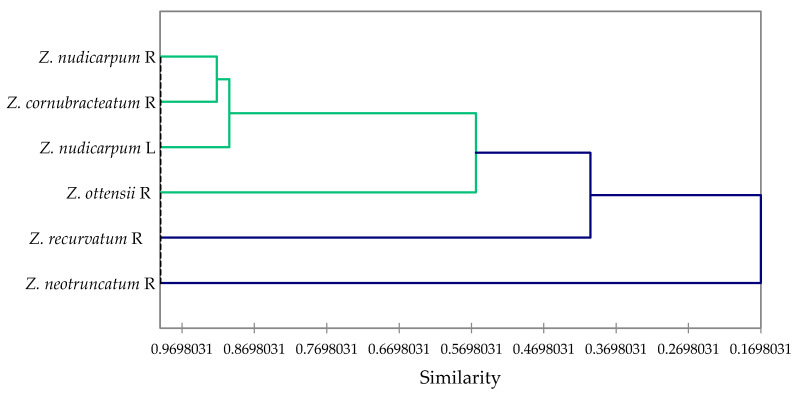
Dendrogram obtained from agglomerative hierarchical cluster analysis of the *Zingiber* essential oils screened for larvicidal activity.

**Figure 4:**
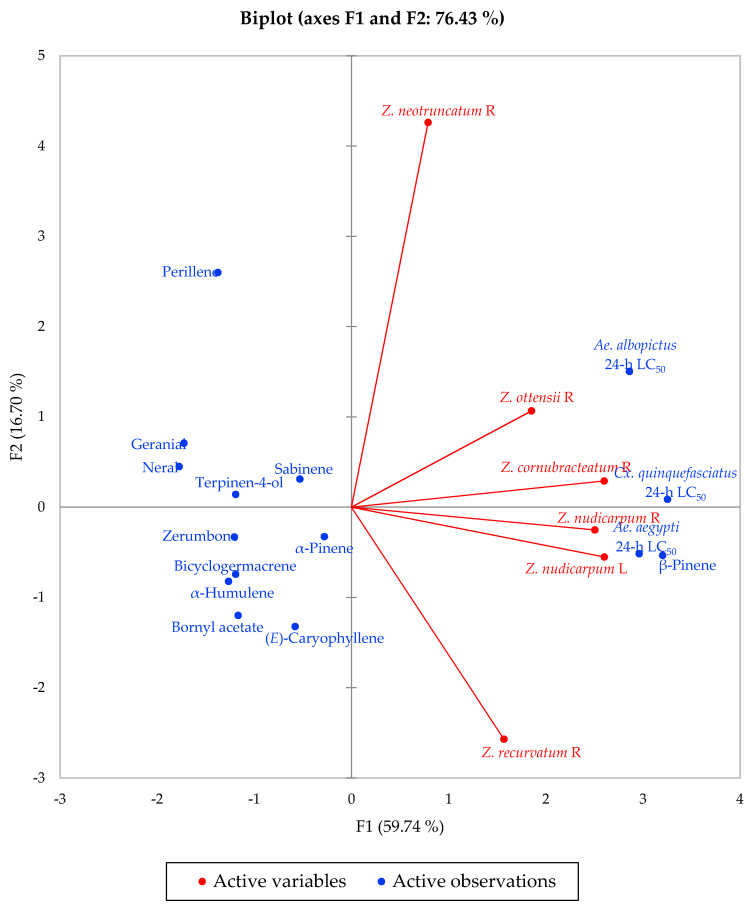
Principal component biplot of PC1 and PC2 scores and loadings indicating the correlations between *Zingiber* essential oil major components and larvicidal activities.

Multivariate analysis of the concentrations of the major components in the essential oils that were used for larvicidal activity screening (α-pinene, sabinene, β-pinene, perillene, terpinene-4-ol, neral, geranial, bornyl acetate, (*E*)-caryophyllene, α-humulene, bicyclogermacrene, and zerumbone), along with their 24-h larvicidal activities, reveals the correlation between activity and composition. The agglomerative hierarchical cluster (AHC) analysis (Figure 3) shows a cluster of largely active essential oils (*Z. nudicarpum* rhizome, *Z. cornubracteatum* rhizome, and *Z. nudicarpum* leaf), along with a marginally active essential oil (*Z. ottensii* rhizome), and two less active essential oils (*Z. recurvatum* rhizome and *Z. neotruncatum* rhizome). The concentrations of β-pinene and, to some extent, α-pinene, are largely responsible for the larvicidal activities of *Zingiber* essential oils, as seen in the principal component analysis (PCA) (Figure 4). Consistent with this correlation, β-pinene has shown larvicidal activity against *Ae. Aegypti,* with LC_50_ values ranging from 12.1 to 42.5 μg/mL [52]. β-Pinene has also shown larvicidal activities against *Ae. albopictus* (LC_50_ of 47.33 and 42.39 μg/mL for (+)-β-pinene and (−)-β-pinene, respectively) [53] and *Cx. quinquefasciatus* (LC_50_ = 19.6 μg/mL) [54]. α-Pinene and sabinene have also shown larvicidal activity against *Ae. aegypti* [52], *Ae. albopictus* [53,55,56], and *Cx. quinquefasciatus* [57,58].

Several *Zingiber* essential oils have been screened for mosquito larvicidal activity. Consistent with the correlation of α- and β-pinene with *Zingiber* essential oil larvicidal activities, *Z. nimmonii* rhizome essential oil, with no α-pinene and only low β-pinene (0.8%), showed relatively marginal larvicidal activity against *Ae. aegypti* and *Cx. quinquefasciatus* (LC_50_ values of 44.5 and 48.3 μg/mL, respectively) [59]. Similarly, *Z. cernuum* rhizome essential oil, with 1.6% α-pinene and 1.2% β-pinene, showed relatively marginal larvicidal activities against *Ae. aegypti* (LC_50_ 44.9 μg/mL), *Ae. albopictus* (LC_50_ 55.8 μg/mL), or *Cx. quinquefasciatus* (LC_50_ 48.4 μg/mL) [60], and the larvicidal activity of *Z. zerumbet* rhizome essential oil (0.8% α-pinene, 0.1% β-pinene) showed larvicidal activities against *Ae. albopictus* and *Cx. quinquefasciatus* with LC_50_ = 55.8 and 33.3 μg/mL, respectively [61]. Finally, *Z. officinale* rhizome oil showed relatively weak activity on *Cx. quinquefasciatus* larvae (LC_50_ = 50.8 μg/mL) [62]. Although the *Z. officinale* rhizome essential oil composition was not determined in this study, commercial *Z. officinale* oil (doTERRA International) contains 4.0% α-pinene and 0.4% β-pinene. In contrast, *Z. collinsii* rhizome essential oil, with 9.0% α-pinene and 16.3% β-pinene, demonstrated more effective larvicidal activity against *Ae. albopictus* with an LC_50_ of 25.5 μg/mL [63].

### 2.3. Antimicrobial Activity

Several of the essential oils from *Zingiber* species were tested for antibacterial activity against a panel of Gram-positive (*Enterococcus faecalis*, *Staphylococcus aureus*, and *Bacillus cereus*), and Gram-negative (*Escherichia coli*, *Pseudomonas aeruginosa*, and *Salmonella enterica*) bacteria, and for anticandidal activity against *Candida albicans* (Table 6). The essential oils generally showed good to excellent activity against the Gram-positive organisms compared to Gram-negative. It has frequently been noted that Gram-positive bacteria demonstrate a higher susceptibility to essential oils than do Gram-negative organisms [64,65,66]. This phenomenon has been attributed to the existence of cell wall lipopolysaccharides in the Gram-negative bacteria, which can inhibit the hydrophobic essential oil constituents from diffusing into the cells [67,68]. *Candida albicans* was also relatively sensitive to the *Zingiber* essential oils.

The essential oil with the best overall antimicrobial activity was *Z. nudicarpum* rhizome essential oil from Pù Hoạt Nature Reserve with MIC < 10 μg/mL against all three Gram-positive organisms and MIC = 16 μg/mL against *P. aeruginosa* and *C. albicans*. It is difficult to correlate essential oil composition with antimicrobial activity, however. The rhizome essential oil of *Z. nudicarpum* was rich in α-pinene (18.7%) and β-pinene (58.3%). The antimicrobial activities of α-pinene and β-pinene have ranged from excellent to inactive against *E. faecalis*, *S. aureus*, *B. cereus*, or *C. albicans* [69,70,71]. However, the presence of these two compounds as major components is not enough to impart good antimicrobial activity. The leaf essential oil of *Z. recurvatum* and the leaf and stem essential oils of *Z. cornubracteatum* from Bến En National Park were also rich in α-pinene (16.3%, 10.1%, and 9.9%, respectively) and β-pinene (71.6%, 67.3%, and 66.8%), but these essential oils showed significantly lower antimicrobial activity. There are likely synergistic and/or antagonistic effects of minor components responsible for the activities.

## 3. Materials and Methods 

### 3.1. Plant Material

*Zingiber* plants were collected from several locations in north-central Vietnam (Table 1, Figure 1). The fresh plant materials (leaves, stems, and/or rhizomes; 2.0 kg each) were immediately chopped and hydrodistilled using a Clevenger apparatus for 4 h to give the essential oils.

### 3.2. Gas Chromatographic Analysis

Gas chromatography with flame ionization detection (GC-FID) was carried out as previously described [72]: Agilent Technologies HP 7890A Plus Gas chromatograph (Santa Clara, CA, USA), flame ionization detector (FID), HP-5ms column (30 m × 0.25 mm, film thickness 0.25 μm, Agilent Technologies), H_2_ carrier gas (1 mL/min), injector temperature = 250 °C, detector temperature = 260 °C, column temperature program: 60 °C (2 min hold), increase to 220 °C (4 °C /min), 220 °C (10 min hold), inlet pressure = 6.1 kPa, split mode injection (10:1) split ratio), 1.0 μL injection volume. 

Gas chromatography–mass spectrometry (GC-MS) was carried out as previously described [72]: Agilent Technologies HP 7890A Plus Chromatograph (Santa Clara, CA, USA), HP-5ms (30 m × 0.25 mm, film thickness 0.25 μm) column, HP 5973 MSD mass detector, He carrier gas (1 mL/min), MS ionization voltage = 70 eV, emission current = 40 mA, acquisitions range = 35–350 amu, sampling rate = 1.0 scan/s. The GC operating conditions were the same as those used for GC-FID. The chemical components of the essential oils were identified based on their retention indices (RI) based on a series of *n*-alkanes, co-injection with pure compounds when available (Sigma-Aldrich, St. Louis, MO, USA) or identified essential oil components, MS library search (NIST 17 and Wiley Version 10) and by comparing with the literature MS fragmentation [73]. The relative concentrations (%) of the components were calculated based on the GC peak area (FID response) without correction factors. The measurements were carried out in triplicate.

### 3.3. Mosquito Larvicidal Screening

Larvae of *Ae. aegypti*, *Ae. albopictus*, and *Cx. quinquefasciatus* were raised in the laboratory as previously described [74]. *Aedes aegypti* larvae were reared from eggs (Institute of Biotechnology, Vietnam Academy of Science and Technology). Adults of *Culex quinquefasciatus* and *Aedes albopictus* were collected in Hoa Khanh Nam ward, Lien Chieu district, Da Nang city (16°03′14.9″ N, 108°09′31.2″ E) and were maintained as described previously [74]. Eggs were hatched and the larvae reared as previously described [74].

Fourth instar larvae of each mosquito species were used for the larvicidal assays, which were carried out as previously described [74]: 250-mL beakers, 150 mL of water, and 20 larvae, aliquots of the *Zingiber* essential oils dissolved in EtOH (1% stock solution) were added to give final concentrations of 100, 50, 25, 12.5, 6, and 3 μg/mL; EtOH only was the negative control, permethrin was the positive control, mortality was recorded after 24 and 48 h of exposure, experiments were carried out at 25 ± 2 °C, assays were carried out in quadruplicate. The larvicidal data were subjected to log-probit analysis [75] to obtain LC_50_ values, LC_90_ values and 95% confidence limits using Minitab^®^ 19.2020.1 (Minitab, LLC, State College, PA, USA).

All procedures involving vertebrates (mice, chicks) were carried out in accordance with the “Guideline for the Care and Use of Laboratory Animals” which was approved by the Medical-Biological Research Ethics Committee of Duy Tan University (DTU/REC2020/NHH01), Vietnam.

### 3.4. Antimicrobial Screening

Antimicrobial activity of *Zingiber* essential oils was carried out on three Gram-negative organisms, *Salmonella enterica* (ATCC 13076), *Pseudomonas aeruginosa* (ATCC 27853), and *Escherichia coli* (ATCC 25922); three Gram-positive organisms, *Bacillus cereus* (ATCC 14579), *Enterococcus faecalis* (ATCC 299212), and *Staphylococcus aureus* (ATCC 25923); and the pathogenic yeast, *Candida albicans* (ATCC 10231), using the microbroth dilution assay as previously described [72]. Dilutions were formulated from 16,384 to 2 μg/mL in sterile distilled water and pipetted into 96-well microplates. Bacteria were grown in tryptic soy broth or Mueller–Hinton broth (double-strength), fungi were grown in Sabouraud dextrose broth (double-strength). Bacteria and fungi were standardized to 5 × 10^5^ CFU/mL for bacteria and 1 × 10^3^ CFU/mL for the yeast. The final lane, containing only serial dilutions of the essential oil without bacteria or yeast, was treated as the positive control. Sterile water (no sample) and media with microorganisms were the negative controls; streptomycin was the positive antibiotic standard; cycloheximide and nystatin served as positive antifungal standards. The plates were incubated at 37 °C for 24 h and the minimum inhibitory concentrations were established as the well with the lowest concentration completely inhibiting microbial growth based on turbidity. The IC_50_ values were determined spectrophotometrically (EPOCH2C spectrophotometer, BioTeK Instruments, Inc Highland Park Winooski, VT, USA) and computed according to the following

$$\%\;\mathrm{inhibition}=\frac{{\mathrm{OD}}_{\mathrm{control}\left(-\right)}-{\mathrm{OD}}_{\mathrm{test}\;\mathrm{agent}}}{{\mathrm{OD}}_{\mathrm{control}\left(-\right)}-{\mathrm{OD}}_{\mathrm{control}\left(+\right)}}\times 100\%$$

$${\mathrm{IC}}_{50}={\mathrm{High}}_{\mathrm{conc}}-\frac{\left({\mathrm{High}}_{\mathrm{inh}\%}-50\%\right)\times \left({\mathrm{High}}_{\mathrm{conc}}-{\mathrm{Low}}_{\mathrm{conc}}\right)}{\left({\mathrm{High}}_{\mathrm{inh}\%}-{\mathrm{Low}}_{\mathrm{inh}\%}\right)}$$

where OD = optical density, control(−) = cells with medium but no antimicrobial agent, test agent is a known concentration of antimicrobial agent, control(+) = culture medium without cells, High_conc_/Low_conc_ = concentration of test agent at high concentration/low concentration and High_inh%_/Low_inh%_ = %inhibition at high concentration/% inhibition at low concentration). The antimicrobial assays were carried out in triplicate.

## 4. Conclusions

There are wide variations in essential oil compositions from the *Zingiber* species in this study, not only between species and tissues, as expected, but also between essential oils from the same species and tissues collected from different locations. This is an important consideration if the essential oils are to be used for agricultural or medicinal uses, but also if commercialization is considered. The monoterpenes α-pinene and β-pinene seem to be largely responsible for the mosquito larvicidal activities observed. It is worth investigating whether *Zingiber* or other essential oils rich in these components are viable alternatives for vector control. The presence of α-pinene and β-pinene cannot explain the antimicrobial activities of *Zingiber* essential oils, and synergistic or antagonistic interactions likely contribute. Nevertheless, several *Zingiber* essential oils have shown excellent antimicrobial activity and should be investigated further for controlling Gram-positive bacterial and yeast infections.

## Figures and Tables

**Table 1:** Plant collection and hydrodistillation details of *Zingiber* species from north-central Vietnam.

*Zingiber* Species	Vietnamese Name	Collection Site Coordinates; Elevation	Collection Month Year	Voucher Number	Plant Part	% Yield (*v*/*w*)
*Zingiber cornubraceatum* Triboun & K. Larsen	Gừng lá bắc cựa	Khe Kèm water fall, Pù Mát National Park 19°58′14″ N, 104°48′20″ E; 280 m	September 2019	830	Leaf Rhizome	0.12 0.15
		Xuân Lý commune, Như Thanh District, Bến En National Park 19°39′09″ N, 105°30′24″ E; 80 m	October 2019	832	Leaf Stems Rhizome	0.14 0.10 0.17
		Tri Lễ commune, Quế Phong District, Pù Hoạt Nature Reserve 19°38′57″ N, 104°40′55″ E; 660 m	September 2018	735	Leaf Rhizome	0.10 0.12
*Zingiber neotruncatum* T.L. Wu, K. Larsen & Turland	Gừng lá mới	Đồng Văn commune, Quế Phong district, Pù Hoạt Nature Reserve 19°53′58″ N, 105°07′45″ E; 315 m	October 2018	746	Rhizome	0.26
*Zingiber nitens* M.F. Newman	Gừng lá sáng bóng	Vũ Quang National Park 18°17′13″ N, 105°26′12″ E; 180 m	September 2018	738	Leaf Stems	0.21 0.45
*Zingiber nudicarpum* D.Fang	Gừng quả trần	Thông Thụ commune, Quế Phong district, Pù Hoạt Nature Reserve 19°49′5″ N, 104°55′35″ E; 460 m	April 2019	760	Leaf Stems Rhizome	0.12 0.08 0.15
		Bạch Mã National Park 16°13′44″ N, 107°53′54″ E; 350 m	April 2018	778	Leaf Stems	0.15 0.10
		Nam Đông District, Thừa Thiên Huế province Nam Đông District, Thừa Thiên Huế province 16°13′9″ N, 107°43′28″ E; 110 m	July 2019	777	Leaf Stems Rhizome	0.14 0.09 0.18
*Zingiber ottensii* Valeton	Gừng ottensi	Tri Lễ commune, Quế Phong district, Pù Hoạt Nature Reserve 19°39′05″ N, 105°40′59″ E; 640 m	April 2019	772	Leaf Roots	0.18 0.25
*Zingiber recurvatum* S.Q. Tong & Y.M. Xia	Gừng lá bắc cong	Tri Lễ commune, Quế Phong district, Pù Hoạt Nature Reserve 19°39′37″ N, 104°40′29″ E; 640 m	July 2019	792	Leaf Rhizome	0.14 0.19
*Zingiber vuquangensis* Ly N.S., Le T.H., Trinh T.H., Nguyen V.H., Do N.D.	Gừng vũ quang	Nậm Nhong commune, Quế Phong district, Pù Hoạt Nature Reserve 19°30′31″ N, 104°43′13″ E; 590 m	April 2019	771	Leaf Stems Rhizome	0.15 0.10 0.14

**Table 2:** Chemical compositions of essential oils of *Zingiber* species from north-central Vietnam.

**RI_calc_**	**RI_db_**	**Compounds**	***Z. cornubracteatum* (Pù Hoạt) Leaf**		***Z. cornubracteatum* (Pù Hoạt) Rhizome**	***Z. cornubracteatum* (Pù Mát) Leaf**	***Z. cornubracteatum* (Pù Mát) Rhizome**	***Z. cornubracteatum* (Bến En) leaf**	***Z. Cornubracteatum* (Bến En) Stem**	***Z. cornubracteatum* (Bến En) Rhizome**	***Z. neotruncatum* Rhizome**	***Z. nitens* Leaf**	***Z. nitens* Rhizome**	***Z. ottensii* Leaf**	***Z. ottensii* Rhizome**
899	894	2-Heptanol	---		---	---	---	---	---	---	---	---	---	---	---
921	923	Tricyclene	---		---	---	---	---	---	---	---	---	0.9	---	---
925	927	α-Thujene	---		---	---	2.1	---	---	2.2	---	---	---	---	0.9
934	933	α-Pinene	5.1		14.5	2.7	8.2	10.1	9.9	9.8	1.9	11.2	10.5	4.0	3.4
943	945	α-Fenchene	---		---	---	0.3	---	---	0.3	---	---	0.9	---	---
951	953	Camphene	---		1.2	---	3.2	0.3	0.3	1.3	0.2	0.3	40.4	---	0.3
952	953	Thuja-2,4(10)diene	---		---	---	---	---	---	---	---	---	---	---	---
971	972	Sabinene	0.8		0.5	0.9	0.7	1.4	1.4	11.9	0.4	1.7	0.2	0.4	21.6
978	978	β-Pinene	20.1		14.8	18.8	8.8	67.3	66.8	33.1	3.1	6.0	1.1	17.1	11.7
981	985	6-Methylhept-5-en-2-one	---		---	---	---	---	---	---	0.3	---	---	---	---
988	991	Myrcene	0.4		0.4	1.8	7.7	1.0	1.0	4.2	0.3	0.4	2.0	0.4	1.3
989	988	Dehydro-1,8-cineole	---		---	---	---	---	---	---	---	---	---	---	---
989	984	2-Pentylfuran	---		---	---	---	---	---	---	---	---	---	---	---
1002	998	Octanal	---		---	---	---	---	---	---	---	---	---	---	---
1004	1108	1,3,8-*p*-Menthatriene	---		---	---	---	---	---	---	---	---	---	---	---
1006	1007	α-Phellandrene	0.8		0.4	1.1	0.4	---	---	1.6	---	---	0.2	---	0.6
1008	1009	δ-3-Carene	0.2		0.1	0.2	5.3	---	---	2.1	---	---	---	---	---
1016	1018	α-Terpinene	---		---	0.2	0.2	---	---	0.8	---	---	---	---	3.5
1018	1022	*m*-Cymene	---		---	---	0.1	---	---	---	---	---	---	---	---
1023	1025	*p*-Cymene	0.4		0.5	0.3	3.9	---	---	2.7	---	---	---	---	2.1
1034	1028	Limonene	0.6		1.0	1.2	5.1	1.2	1.2	3.4	0.2	0.9	4.6	0.8	0.9
1035	1031	β-Phellandrene	0.5		0.9	0.8	2.2	0.3	0.3	0.5	---	0.2	---	0.2	0.3
1035	1030	1,8-Cineole	---		10.4	---	6.1	0.2	0.2	2.5	0.7	---	---	---	4.3
1036	1034	(*Z*)-β-Ocimene	0.3		---	---	---	---	---	---	---	0.2	0.9	0.3	---
1046	1045	(*E*)-β-Ocimene	0.6		0.4	0.4	0.4	0.1	0.1	0.8	1.2	2.1	12.7	2.1	0.9
1057	1057	γ-Terpinene	0.3		0.4	0.4	0.7	0.2	0.2	2.4	---	---	0.1	---	5.5
1067	1063	1-Octanol	---		---	---	---	---	---	---	---	---	---	---	---
1068	1067	*cis*-Linalool oxide (furanoid)	---		0.3	---	---	---	---	---	---	---	---	---	---
1072	1065	*cis*-Sabinene hydrate	---		---	---	---	---	---	---	---	---	---	---	0.3
1084	1086	Terpinolene	0.1		---	0.3	---	---	---	0.7	---	---	0.8	---	1.1
1084	1084	*trans*-Linalool oxide (furanoid)	---		0.3	---	---	---	---	---	---	---	---	---	---
1087	1083	Fenchone	---		---	---	---	---	---	---	---	---	0.2	---	---
1088	1089	*p*-Cymenene	---		---	---	---	---	---	---	---	---	---	---	---
1099	1102	Perillene	---		---	---	0.3	0.1	0.1	---	51.3	---	---	---	---
1099	1097	2-Nonanol	---		---	---	0.3	---	---	---	---	---	---	---	---
1100	1101	Linalool	0.5		31.0	-	11.6	0.2	0.2	0.4	---	0.4	---	---	0.2
1100	1104	Rosefuran	---		---	---	---	---	---	---	0.2	---	---	---	---
1104	1098	*trans*-Sabinene hydrate	---		0.3	---	---	---	---	---	---	---	---	---	0.2
1105	1107	Nonanal	---		---	---	---	---	---	---	---	---	---	---	---
1112	1116	(*E*)-4,8-Dimethylnona-1,3,7-triene	---		---	0.1	0.2	---	---	---	---	---	---	---	---
1118	1114	*endo*-Fenchol	---		---	---	0.3	---	---	0.2	---	---	---	---	---
1118	1118	*cis-p*-Menth-2-en-1-ol	---		---	---	---	---	---	---	---	---	---	---	0.4
1120	1128	*allo*-Ocimene	---		---	---	---	---	---	---	---	---	---	---	---
1124	1118	*exo*-Fenchol	---		0.3	---	---	---	---	---	---	---	---	---	---
1128	1122	α-Campholenal	---		---	---	0.1	---	---	---	---	---	---	---	---
1135	1136	*trans-p*-Menth-2-en-1-ol	---		---	---	---	---	---	---	---	---	---	---	0.3
1137	1135	Nopinone	---		---	---	---	---	---	---	---	---	---	---	---
1141	1135	*trans*-Pinocarveol	---		---	---	---	---	---	0.2	---	---	---	---	---
1144	1140	*trans*-Verbenol	---		0.8	---	---	---	---	---	---	---	---	---	---
1146	1149	Camphor	---		---	---	---	---	---	---	---	---	---	---	---
1148	1165	Lavandulol	---		---	---	---	---	---	---	0.2	---	---	---	---
1159	1167	Benzyl acetate	---		---	---	---	---	---	---	---	---	---	---	---
1162	1160	Pinocarvone	---		---	---	---	---	---	---	---	---	---	---	---
1167	1165	*iso*-Neral	---		---	---	---	---	---	---	0.7	---	---	---	---
1169	1162	δ-Terpineol	---		---	---	---	---	---	---	---	---	---	---	---
1170	1166	*p*-Mentha-1,5-dien-8-ol	---		---	---	---	---	---	---	---	---	---	---	---
1171	1165	Borneol	---		3.2	---	0.7	---	---	0.1	---	---	0.8	---	0.5
1180	1180	Terpinen-4-ol	0.1		0.7	---	0.5	---	---	1.4	0.1	---	---	---	17.1
1185	1179	*p*-Cymen-8-ol	---		---	---	---	---	---	---	---	---	---	---	---
1185	1185	*iso*-Geranial	---		---	---	---	---	---	---	1.1	---	---	---	---
1194	1195	α-Terpineol	---		0.6	---	0.2	---	---	0.1	---	---	---	---	0.9
1194	1195	Myrtenal	---		---	---	---	---	---	---	---	---	---	---	---
1194	1193	(4*Z*)-Decenal	---		---	---	---	---	---	---	---	---	---	---	---
1195	1194	Myrtenol	1.0		0.2	---	---	---	---	---	---	---	---	---	---
1198	1195	*cis*-Piperitol	---		---	---	---	---	---	---	---	---	---	---	0.1
1205	1204	Verbenone	---		---	---	---	---	---	---	---	---	---	---	---
1206	1206	Decanal	---		---	---	---	---	---	---	---	---	---	---	---
1209	1207	*trans*-Piperitol	---		---	---	---	---	---	---	---	---	---	---	0.2
1216	1218	*endo*-Fenchyl acetate	---		---	---	0.6	---	---	0.5	---	---	3.3	---	---
1217	1215	*trans*-Carveol	---		---	---	---	---	---	---	---	---	---	---	---
1220	1217	β-Cyclocitral	---		---	---	---	---	---	---	---	---	---	---	---
1222	1227	Nerol	---		---	---	---	---	---	---	---	---	---	---	---
1224	1223	Citronellol	---		---	---	---	---	---	---	---	---	---	---	---
1227	1232	Thymyl methyl ether	---		---	---	---	---	---	---	---	---	---	---	---
1236	1235	Neral	---		---	---	---	---	---	---	12.3	---	---	---	---
1241	1238	Cuminal	---		---	---	---	---	---	---	---	---	---	---	---
1242	1239	Carvone	---		---	---	---	---	---	---	---	---	---	---	---
1248	1249	Geraniol	---		---	---	0.2	---	---	---	0.3	---	---	---	---
1265	1264	Geranial	---		---	---	---	---	---	---	17.0	---	---	---	---
1271	1266	1-Decanol	---		---	---	0.2	---	---	---	---	---	---	---	---
1281	1287	*iso*-Bornyl acetate	---		---	---	---	---	---	---	---	---	---	---	---
1284	1285	Bornyl acetate	0.2		2.8	---	7.9	---	---	2.5	---	---	14.5	---	0.3
1285	1291	Safrole	---		---	---	---	---	---	---	---	---	---	---	---
1293	1290	Dihydroedulan IIA	---		---	---	---	---	0.2	---	---	---	---	0.3	---
1295	1298	Carvacrol	---		---	---	---	---	---	---	---	---	---	---	---
1295	1293	Methyl myrtenate	---		---	---	---	---	---	---	---	---	---	---	---
1307	1295	*trans*-Sabinyl acetate	---		---	---	---	---	---	0.2	---	---	---	---	---
1330	1324	Methyl geranate	---		---	---	0.3	---	---	---	0.2	---	---	---	---
1330	1332	Bicycloelemene	---		---	---	---	---	---	---	---	---	---	---	---
1330	1335	δ-Elemene	1.4		---	1.5	---	0.4	0.5	---	---	---	---	0.2	---
1333	1325	Myrtenyl acetate	0.1		0.5	---	2.0	---	---	0.7	---	---	---	---	---
1342	1349	α-Cubebene	0.1		---	---	---	---	---	---	---	---	---	---	---
1345	1346	α-Terpinyl acetate	---		---	---	---	---	---	---	---	---	---	---	---
1364	1367	Cyclosativene	---		---	---	---	---	---	---	---	---	---	---	---
1364	1372	*iso*-Ledene	---		---	---	---	---	---	---	---	---	---	---	---
1368	1373	α-Ylangene	---		---	---	---	---	---	---	---	---	---	---	---
1374	1375	α-Copaene	0.2		---	0.4	---	---	---	---	---	---	---	3.2	---
1375	1380	Geranyl acetate	---		---	---	---	---	---	---	0.6	---	---	---	---
1377	1383	*cis*-β-Elemene	---		---	---	---	---	---	---	---	---	---	---	---
1377	1365	Eugenol	---		---	---	---	---	---	---	---	---	---	---	---
1382	1382	β-Bourbonene	---		---	---	---	---	---	---	---	---	---	---	---
1386	1387	β-Cubebene	0.4		---	0.5	---	---	---	---	---	---	---	---	---
1387	1390	*trans*-β-Elemene	0.2		---	0.5	---	3.9	4.4	0.7	---	0.8	0.2	17.0	0.3
1400	1403	Methyl eugenol	---		---	---	---	---	---	---	---	---	---	---	---
1403	1405	Sesquithujene	---		---	---	---	---	---	---	---	0.1	---	---	---
1403	1408	(*Z*)-Caryophyllene	---		---	---	---	---	---	---	---	---	---	---	---
1405	1406	α-Gurjunene	---		---	0.3	---	---	---	0.2	---	---	---	0.6	---
1405	1415	β-Maaliene	---		---	---	---	---	---	---	---	---	---	---	---
1415	1416	*cis*-α-Bergamotene	---		---	---	---	---	---	---	---	---	---	---	---
1419	1417	(*E*)-Caryophyllene	8.9		0.4	13.9	1.7	1.8	2.1	0.6	1.5	---	---	28.0	0.7
1427	1432	γ-Elemene	0.3		---	---	---	0.2	0.3	0.1	---	---	---	---	---
1428	1424	2,5-Dimethoxy-*p*-cymene	---		---	---	---	---	---	---	---	---	---	---	---
1428	1430	β-Copaene	---		---	---	---	---	---	---	---	---	---	---	---
1428	1438	α-Maaliene	---		---	---	---	---	---	---	---	---	---	---	---
1431	1416	α-Santalene	---		---	---	---	---	---	---	---	---	---	---	---
1431	1432	*trans*-α-Bergamotene	---		---	---	---	---	---	---	---	---	---	---	---
1433	1437	α-Guaiene	---		---	---	---	---	---	---	---	---	---	---	---
1437	1438	Aromadendrene	*0.3*		---	0.3	---	0.2	---	---	---	---	---	0.1	---
1440	1440	(Z)-β-Farnesene	---		---	---	---	---	---	---	---	0.3	---	---	---
1444	1431	β-Gurjunene	0.3		---	0.6	---	---	---	---	---	---	---	---	---
1445	1445	Myltayl-4(12)-ene	---		---	---	---	---	---	---	---	---	---	---	---
1446	1453	Geranyl acetone	---		---	---	---	---	---	---	---	---	---	---	---
1447	1453	*trans*-Muurola-3,5-diene	---		---	---	---	---	---	---	---	---	---	---	---
1450	1452	(*E*)-β-Farnesene	---		---	---	---	---	---	---	---	0.2	---	---	---
1452	1457	Prezizaene	---		---	---	---	---	---	---	---	---	---	---	---
1454	1454	α-Humulene	0.7		---	1.0	0.3	0.2	0.3	---	---	---	---	3.0	3.8
1458	1457	*allo*-Aromadendrene	---		---	---	---	---	---	---	---	---	---	---	---
1460	1461	*cis*-Cadina-1(6),4-diene	---		---	---	---	---	---	---	---	---	---	---	---
1470	1472	*trans*-Cadina-1(6),4-diene	---		---	0.7	---	---	---	---	---	---	---	---	---
1471	1476	β-Chamigrene	---		---	---	---	---	---	---	---	---	---	---	---
1471	1475	γ-Gurjunene	---		---	---	---	---	---	---	---	---	---	---	---
1472	1482	γ-Curcumene	---		---	---	---	---	---	---	---	---	---	---	---
1473	1478	γ-Muurolene	0.3		---	0.5	0.1	---	---	---	---	---	---	---	---
1474	1476	Selina-4,11-diene	---		---	---	---	---	---	---	---	---	---	---	---
1477	1482	α-Amorphene	---		---	---	---	---	---	---	---	---	---	---	---
1477	1479	*ar*-Curcumene	---		---	---	---	---	---	---	---	5.2	0.2	---	---
1477	1487	(*E*)-β-Ionone	---		---	---	---	---	---	---	---	---	---	---	---
1478	1483	*trans*-β-Bergamotene	---		---	---	---	---	---	---	---	---	---	---	---
1479	1464	9-*epi*-(*E*)-Caryophyllene	0.4		---	0.8	---	0.1	0.2	---	---	0.2	---	0.2	---
1480	1480	Germacrene D	1.6		---	13.7	0.8	0.7	0.8	0.2	---	0.7	---	2.5	---
1481	1488	δ-Selinene	---		---	---	---	---	---	---	---	---	---	---	---
1482	1492	*cis*-β-Guaiene	---		---	---	---	---	---	---	---	---	---	---	---
1483	1487	Aristolochene	---		---	---	---	---	---	---	---	---	0.3	---	---
1488	1492	β-Selinene	0.3		---	0.4	---	0.5	0.6	---	---	---	---	0.8	---
1489	1496	Valencene	---		---	---	---	---	---	---	---	---	---	0.4	---
1490	1490	γ-Amorphene	---		---	---	---	---	---	---	---	---	---	---	---
1491	1493	α-Zingiberene	---		---	---	---	---	---	---	---	17.4	1.6	---	---
1494	1498	α-Selinene	---		---	---	---	---	---	---	---	---	---	---	---
1494	1497	Bicyclogermacrene	7.4		---	18.9	0.5	2.7	3.0	0.4	0.9	1.5	---	1.2	---
1496	1500	α-Muurolene	---		---	---	---	---	---	---	---	---	---	---	---
1496	1506	α-Bulnesene	---		---	---	---	---	---	---	---	---	---	---	---
1497	1502	*trans*-β-Guiaene	---		---	---	---	---	---	---	---	---	---	---	---
1500	1500	*n*-Pentadecane	---		---	---	---	---	---	---	0.2	---	---	---	---
1501	1505	(*E*,*E*)-α-Farnesene	---		---	0.9	0.2	0.5	0.6	0.2	---	---	---	1.1	---
1501	1495	γ-Amorphene	---		---	---	---	---	---	---	---	---	---	---	---
1502	1496	Viridiflorene	---		---	---	---	---	---	---	---	---	---	---	---
1503	1509	β-Curcumene	---		---	---	---	---	---	---	---	---	---	---	---
1506	1508	β-Bisabolene	---		---	---	0.2	---	---	0.2	1.2	3.7	0.2	---	---
1511	1512	γ-Cadinene	0.3		---	0.5	---	---	0.2	---	---	---	---	---	---
1511	1511	δ-Amorphene	---		---	0.4	---	0.2	0.2	---	---	---	---	---	---
1513	1519	Cubebol	---		---	---	---	---	---	---	---	---	---	---	---
1516	1518	δ-Cadinene	0.6		-	0.9	0.2	0.2	0.2	---	---	---	---	2.2	---
1517	1520	7-*epi*-α-Selinene	---		---	---	---	---	---	---	---	---	---	---	---
1517	1521	Zonarene	---		---	---	---	---	---	---	---	---	---	---	---
1519	1521	*trans*-Calamenene	---		---	---	---	---	---	---	---	---	---	---	---
1520	1528	(*E*)-γ-Bisabolene	---		---	---	---	---	---	---	---	0.2	---	---	---
1523	1521	Eugenyl acetate	---		---	---	---	---	---	---	---	---	---	0.2	0.1
1524	1521	β-Sesquiphellandrene	---		---	---	---	---	---	---	---	10.1	0.6	---	---
1529	1528	*cis*-Calamene	---		---	---	---	---	---	---	---	---	---	---	---
1530	1536	*trans*-Cadina-1,4-diene	---		---	---	---	---	---	---	---	---	---	---	---
1535	1537	α-Cadinene	---		---	---	---	---	---	---	---	---	---	---	---
1539	1544	α-Calacorene	---		---	---	---	---	---	---	---	---	---	---	---
1546	1549	α-Elemol	0.2		---	0.2	---	---	---	0.2	---	---	---	0.2	0.4
1557	1559	Germacrene B	0.3		---	0.5	---	0.2	0.3	0.3	---	---	---	---	---
1558	1561	(*E*)-Nerolidol	23.0		---	2.7	8.4	0.9	1.1	0.3	0.1	10.0	1.1	0.5	---
1560	1564	β-Calacorene	---		---	---	---	---	---	---	---	---	---	---	---
1564	1568	Palustrol	---		---	---	---	---	---	---	---	---	---	---	---
1568	1571	Maaliol	---		---	---	---	---	---	---	---	---	---	---	---
1570	1573	(3*E*,7*E*)-4,8,12-Trimethyltrideca-1,3,7,11-tetraene	---		---	---	---	---	---	---	---	---	---	---	---
1575	1576	Spathulenol	2.5		---	1.4	---	0.1	0.2	---	0.4	0.6	---	0.2	---
1575	1582	Neryl isovalerate	---		---	---	---	---	---	---	---	---	---	---	---
1575	1574	Germacrene D-4-ol	0.2		---	---	---	---	---	---	---	0.9	---	---	---
1582	1577	Caryophyllene oxide	2.2		---	2.0	0.6	---	0.2	0.3	0.3	---	0.1	1.2	0.4
1582	1582	*ar*-Turmerol	---		---	---	---	---	---	---	---	0.2	---	---	---
1583	1570	Dendrolasin	---		---	---	---	---	---	---	---	---	---	---	---
1583	1585	*epi*-Globulol	---		---	---	---	---	---	---	---	---	---	---	---
1587	1590	Globulol	---		---	---	---	---	---	---	---	---	---	---	---
1590	1581	Clovenol	---		---	---	---	---	---	---	---	---	---	---	---
1592	1594	Salvial-4(14)en-1-one	---		---	---	---	---	---	---	---	---	---	---	---
1593	1592	Viridiflorol	---		---	---	---	---	---	---	---	---	---	---	---
1593	1593	Scapanol ^g^	---		---	0.3	---	---	---	---	---	---	---	---	---
1594	1600	Guaiol	0.5		---	0.2	---	0.1	0.2	0.3	---	---	---	---	---
1594	1601	*trans*-β-Elemenone	---		---	---	---	---	---	---	---	---	---	---	---
1596	1599	Cubeban-11-ol	---		---	---	---	---	---	---	---	---	---	---	---
1599	1592	Humulene epoxide I	---		---	---	---	---	---	---	---	---	---	---	1.2
1600	1609	Rosifoliol	---		---	---	---	---	---	---	---	---	---	---	---
1604	1607	5,7-di-*epi*-Eudesmol	---		---	---	---	---	---	---	---	---	---	---	---
1604	1602	Ledol	0.3		---	0.3	---	---	---	---	---	---	---	---	---
1613	1618	1,10-di-*epi*-Cubenol	---		---	---	---	---	---	---	---	---	---	---	---
1616	1613	Humulene epoxide II	---		---	---	0.2	---	---	---	---	---	---	---	0.6
1616	1611	Zingiberenol	---		---	---	---	---	---	---	---	7.2	0.2	---	---
1618	1623	Humulane-1,6-dien-3-ol	---		---	---	---	---	---	---	---	---	---	---	---
1620	1622	10-*epi*-γ-Eudesmol	---		---	0.3	---	---	---	---	---	---	---	---	---
1625	1631	1-*epi*-Cubenol	---		---	0.2	---	---	---	---	---	---	---	---	---
1627	1618	*epi*-Cedrol	---		---	---	---	---	---	---	---	---	---	---	---
1628	1622	Alismol	0.4		---	---	---	---	---	---	---	---	---	---	---
1630	1630	γ-Eudesmol	---		---	---	---	---	---	---	---	---	---	---	0.2
1632	1631	Caryophylla-4(12),8(13)-dien-5α-ol	---		---	---	---	---	---	---	---	---	---	---	---
1634	1632	α-Acorenol	---		---	---	---	---	---	---	---	3.3	0.2	---	---
1636	1639	Caryophylla-4(12),8(13)-dien-5β-ol	---		---	---	---	---	---	---	---	---	---	---	---
1640	1638	τ-Cadinol	0.9		---	0.2	---	---	---	---	---	---	---	---	---
1640	1643	Cubenol	---		---	---	---	---	---	---	---	---	---	---	---
1642	1644	τ-Murrolol	---		---	0.4	---	---	---	---	---	---	---	---	---
1645	1644	α-Muurolol (=δ-Cadinol)	---		---	0.2	---	---	---	---	---	---	---	---	---
1651	1649	β-Eudesmol	---		---	0.3	0.3	---	---	---	---	---	---	---	0.3
1652	1652	α-Cadinol	0.5		---	1.0	---	---	0.1	---	---	0.4	---	0.3	---
1652	1647	1,2-Diacetoxy-4-allylbenzene	---		---	---	---	---	---	---	---	---	---	---	---
1653	1652	α-Eudesmol	---		---	---	---	---	---	---	---	---	---	---	0.3
1657	1658	Selin-11-en-4α-ol	---		---	---	---	---	---	---	---	---	---	---	---
1662	1658	*neo*-Intermedeol	0.5		---	---	---	---	0.1	-	---	0.7	0.2	0.2	---
1668	1668	14-Hydroxy-9-*epi*-(*E*)-caryophyllene	---		---	0.2	---	---	---	---	---	---	---	---	---
1670	1675	Cadalene	---		---	---	---	---	---	---	---	---	---	---	---
1671	1676	Mustakone	---		---	---	---	---	---	---	---	---	---	---	---
1679	1681	γ-Bicyclofarnesal	---		---	---	0.5	---	---	0.5	---	---	---	---	---
1681	1685	α-Bisabolol	---		---	---	---	---	---	---	---	4.1	---	---	---
1684	1685	Germacra-4(15),5,10(14)-trien-1α-ol	---		---	---	---	---	---	---	---	---	---	---	---
1684	1683	*epi*-α-Bisabolol	---		---	---	0.1	---	---	---	---	---	---	---	---
1690	1693	Germacrone	---		---	---	---	---	---	---	---	---	---	---	---
1691	1694	Germacra-4(15),5,10(14)-trien-1β-ol	---		---	---	---	---	---	---	---	---	---	---	---
1696	1689	6α-Hydroxygermacra-1(10),4-diene	---		---	---	---	---	---	---	---	0.7	---	---	---
1701	1698	(2*Z*,6*Z*)-Farnesol	---		---	---	---	---	---	---	---	0.9	---	---	---
1702	1690	(Z)-*trans*-α-Bergamotol	---		---	---	---	---	---	---	---	---	---	---	---
1707	1714	Nootkatol	---		---	---	---	---	---	---	---	---	---	---	---
1713	1713	Pentadecanal	0.2		---	---	---	---	---	0.2	---	---	---	---	---
1738	1740	Mint sulfide	1.5		---	1.9	---	---	0.1	---	---	---	---	---	---
1739	1732	Zerumbone	---		---	---	---	---	---	---	---	---	---	---	12.5
1761	1759	Benzyl benzoate	---		---	---	---	---	---	---	---	---	---	---	---
1774	1760	(Z)-Lanceol	---		---	---	---	---	---	---	0.3	---	---	---	---
1799	1809	Ambrial	0.1		---	---	0.9	---	---	3.6	---	---	---	0.3	---
1838	1838	Phytone	---		---	---	---	---	---	---	---	---	---	---	---
1848	1845	6,10,14-Trimethylpentadecan-2-one	1.1		---	---	---	0.1	0.2	---	---	---	---	---	---
1862	1864	Benzyl salicylate	---		---	---	---	---	---	---	---	---	---	---	---
2002	---	(*E*)-15,16-Bisnorlabda-8(17),11-dien-13-one	---		---	---	0.3	---	---	0.2	---	---	---	---	---
2104	2105	(*E*)-Phytol	---		---	0.3	---	0.3	0.4	---	---	---	---	0.2	---
		Monoterpene hydrocarbons	30.2		35.1	29.1	49.3	81.9	81.2	77.8	7.3	23.0	75.3	25.3	54.1
		Oxygenated monoterpenoids	1.9		51.4	0.0	30.8	0.5	0.5	8.8	84.7	0.4	18.8	0.0	24.8
		Sesquiterpene hydrocarbons	24.0		0.4	57.2	4.0	11.8	13.7	2.9	3.6	40.4	3.1	60.5	4.8
		Oxygenated sesquiterpenoids	31.2		0.0	9.9	10.1	1.1	1.9	1.6	1.1	29.0	1.8	2.6	15.9
		Others	2.9		0.0	2.3	1.9	0.4	0.9	4.0	0.5	0.0	0.0	1.0	0.1
		Total identified	90.2		86.9	98.5	96.1	95.7	98.2	95.1	97.2	92.8	99.0	89.4	99.7
**RI_calc_**	**RI_db_**	**Compounds**	***Z. nudicarpum* (Pù Hoạt) leaf**	***Z. nudicarpum* (Pù Hoạt) stem**	***Z. nudicarpum* (Pù Hoạt) rhizome**	***Z. nudicarpum* (Nam Đông) leaf**	***Z. nudicarpum* (Nam Đông) stem**	***Z. nudicarpum* (Nam Đông) rhizome**	***Z. nudicarpum* (Bạch Mã) leaf**	***Z. nudicarpum* (Bạch Mã) stem**	***Z. recurvatum*** **leaf**	***Z. recurvatum* rhizome**	***Z. vuquangensis* leaf**	***Z. vuquangensis* stem**	***Z. vuquangensis* rhizome**
899	894	2-Heptanol	---	---	---	---	0.2	---	---	---	---	---	---	---	---
921	923	Tricyclene	---	---	---	---	---	tr	tr	tr	---	---	---	---	---
925	927	α-Thujene	0.1	---	0.1	0.8	0.1	tr	0.1	tr	0.3	0.3	---	---	---
934	933	α-Pinene	10.9	---	18.7	5.0	10.6	4.0	6.5	6.1	16.3	1.9	11.3	0.5	9.8
943	945	α-Fenchene	---	---	---	---	---	---	---	---	0.2	---	---	---	---
951	953	Camphene	1.6	---	1.3	0.1	0.3	2.1	0.6	0.8	0.4	1.9	0.2	---	0.3
952	953	Thuja-2,4(10)diene	---	---	---	tr	0.2	---	---	---	---	---	---	---	---
971	972	Sabinene	---	---	1.4	2.3	0.1	tr	0.2	0.1	1.2	0.3	1.0	---	0.7
978	978	β-Pinene	34.0	0.5	58.3	26.6	9.0	9.8	0.7	5.6	71.6	4.7	38.5	3.1	45.0
981	985	6-Methylhept-5-en-2-one	---	---	---	---	0.1	---	---	---	---	---	---	---	---
988	991	Myrcene	0.8	---	0.9	0.8	0.4	0.6	0.7	1.2	1.0	2.1	0.5	---	0.7
989	988	Dehydro-1,8-cineole	---	---	---	---	0.1	---	---	---	---	---	---	---	---
989	984	2-Pentylfuran	---	---	---	tr	---	---	---	---	---	---	---	---	---
1002	998	Octanal	---	---	---	---	0.1	---	---	---	---	---	---	---	---
1004	1108	1,3,8-*p*-Menthatriene	---	---	---	---	0.1	---	---	---	---	---	---	---	---
1006	1007	α-Phellandrene	---	---	---	tr	0.1	0.2	0.9	0.6	0.2	0.6	---	---	---
1008	1009	δ-3-Carene	---	---	0.2	---	0.2	3.1	3.7	1.8	---	1.5	---	---	---
1016	1018	α-Terpinene	0.1	---	---	0.2	tr	tr	0.1	tr	---	---	---	---	0.1
1018	1022	*m*-Cymene	---	---	---	---	tr	tr	---	---	---	---	---	---	---
1023	1025	*p*-Cymene	0.2	---	0.4	0.4	6.0	0.9	0.2	0.1	---	0.6	---	---	0.2
1034	1028	Limonene	1.5	---	1.8	2.0	2.1	1.8	1.7	6.0	1.8	2.4	0.9	---	1.9
1035	1031	β-Phellandrene	0.2	---	0.4	0.2	---	0.7	0.3	0.3	0.3	0.6	0.3	---	2.3
1035	1030	1,8-Cineole	0.9	---	0.3	0.9	21.2	6.8	tr	tr	---	0.6	---	---	---
1036	1034	(*Z*)-β-Ocimene	---	---	---	tr	---	---	0.1	0.2	0.2	---	---	---	---
1046	1045	(*E*)-β-Ocimene	---	---	0.7	tr	0.1	---	0.1	0.2	0.6	0.4	---	---	---
1057	1057	γ-Terpinene	0.5	---	0.3	0.8	0.1	0.4	0.1	0.1	0.1	0.6	---	---	0.4
1067	1063	1-Octanol	---	---	---	---	0.3	---	---	---	---	---	---	---	---
1068	1067	*cis*-Linalool oxide (furanoid)	---	---	---	---	---	---	---	---	---	---	---	---	---
1072	1065	*cis*-Sabinene hydrate	---	---	---	---	---	---	---	---	---	---	---	---	---
1084	1086	Terpinolene	0.1	---	0.3	0.2	0.1	0.3	0.1	0.1	---	0.4	---	---	0.4
1084	1084	*trans*-Linalool oxide (furanoid)	---	---	---	---	---	---	---	---	---	---	---	---	---
1087	1083	Fenchone	---	---	---	---	---	---	---	---	---	---	---	---	---
1088	1089	*p*-Cymenene	---	---	---	---	0.1	---	---	---	---	---	---	---	---
1099	1102	Perillene	---	---	---	---	0.1	---	---	---	---	---	---	---	---
1099	1097	2-Nonanol	---	---	---	---	0.1	---	---	---	---	---	---	---	---
1100	1101	Linalool	2.4	11.0	0.5	0.6	0.3	0.5	0.7	1.0	---	3.7	---	---	---
1100	1104	Rosefuran	---	---	---	---	---	---	---	---	---	---	---	---	---
1104	1098	*trans*-Sabinene hydrate	---	---	---	---	---	---	---	---	---	---	---	---	---
1105	1107	Nonanal	---	---	---	0.1	---	---	tr	tr	---	---	---	---	---
1112	1116	(*E*)-4,8-Dimethylnona-1,3,7-triene	0.1	---	---	tr	---	---	---	---	---	0.4	---	---	---
1118	1114	*endo*-Fenchol	---	---	---	---	0.1	0.7	---	---	---	0.2	---	---	---
1118	1118	*cis-p*-Menth-2-en-1-ol	---	---	---	---	---	---	---	---	---	---	---	---	---
1120	1128	*allo*-Ocimene	0.3	---	---	---	---	---	---	---	---	---	---	---	---
1124	1118	*exo*-Fenchol	---	---	---	---	---	---	---	---	---	---	---	---	---
1128	1122	α-Campholenal	---	---	---	tr	0.2	---	---	---	---	---	---	---	---
1135	1136	*trans-p*-Menth-2-en-1-ol	---	---	---	---	---	---	---	---	---	---	---	---	---
1137	1135	Nopinone	---	---	---	---	0.1	---	---	---	---	---	---	---	---
1141	1135	*trans*-Pinocarveol	---	---	---	tr	2.0	0.2	---	---	---	---	---	---	---
1144	1140	*trans*-Verbenol	---	---	---	---	0.4	---	---	---	---	---	---	---	---
1146	1149	Camphor	---	---	---	---	0.1	0.3	0.1	tr	---	---	---	---	---
1148	1165	Lavandulol	---	---	---	---	---	---	---	---	---	---	---	---	---
1159	1167	Benzyl acetate	---	---	---	---	---	---	0.1	tr	---	---	---	---	---
1162	1160	Pinocarvone	---	---	0.2	tr	1.5	0.2	---	---	---	---	0.2	---	0.3
1167	1165	*iso*-Neral	---	---	---	---	---	---	---	---	---	---	---	---	---
1169	1162	δ-Terpineol	---	---	---	---	0.1	---	---	---	---	---	---	---	---
1170	1166	*p*-Mentha-1,5-dien-8-ol	---	---	---	---	0.6	---	---	---	---	---	---	---	---
1171	1165	Borneol	0.2	0.6	---	tr	0.2	4.0	---	---	---	---	---	---	---
1180	1180	Terpinen-4-ol	---	0.2	---	0.1	0.4	0.4	0.1	0.1	0.1	0.1	---	---	---
1185	1179	*p*-Cymen-8-ol	---	---	---	tr	0.3	---	---	---	---	---	---	---	---
1185	1185	*iso*-Geranial	---	---	---	---	---	---	---	---	---	---	---	---	---
1194	1195	α-Terpineol	---	0.2	---	0.1	---	1.6	tr	0.1	---	---	---	---	0.2
1194	1195	Myrtenal	0.2	---	0.3	0.1	2.6	---	---	---	---	---	0.2	---	0.3
1194	1193	(4*Z*)-Decenal	---	---	---	0.1	---	---	---	---	---	---	---	---	---
1195	1194	Myrtenol	---	---	---	---	0.9	---	---	---	---	---	---	---	---
1198	1195	*cis*-Piperitol	---	---	---	---	---	---	---	---	---	---	---	---	---
1205	1204	Verbenone	---	---	---	---	0.3	---	---	---	---	---	---	---	---
1206	1206	Decanal	---	---	---	0.1	---	---	---	0.1	---	0.2	---	---	---
1209	1207	*trans*-Piperitol	---	---	---	---	---	---	---	---	---	---	---	---	---
1216	1218	*endo*-Fenchyl acetate	---	---	0.1	tr	0.5	6.5	---	---	---	0.2	---	---	---
1217	1215	*trans*-Carveol	---	---	---	---	0.2	---	---	---	---	---	---	---	---
1220	1217	β-Cyclocitral	---	---	---	tr	---	---	---	---	---	---	---	---	---
1222	1227	Nerol	---	---	---	---	0.1	---	---	---	---	---	---	---	---
1224	1223	Citronellol	---	---	---	---	0.3	0.4	---	---	---	---	---	---	---
1227	1232	Thymyl methyl ether	---	---	---	---	---	0.4	---	---	---	---	---	---	---
1236	1235	Neral	---	---	---	---	0.1	---	---	---	---	---	---	---	---
1241	1238	Cuminal	---	---	---	---	tr	---	---	---	---	---	---	---	---
1242	1239	Carvone	---	---	---	---	0.2	---	---	---	---	---	---	---	---
1248	1249	Geraniol	---	---	---	---	1.0	0.8	---	---	---	0.3	---	---	---
1265	1264	Geranial	---	---	---	---	0.2	tr	---	---	---	---	---	---	---
1271	1266	1-Decanol	---	---	---	---	---	---	---	---	---	---	---	---	---
1281	1287	*iso*-Bornyl acetate	---	---	---	---	---	---	tr	0.4	---	0.2	---	---	---
1284	1285	Bornyl acetate	---	---	0.3	---	0.4	1.2	0.2	1.1	---	10.4	---	---	---
1285	1291	Safrole	---	---	---	---	---	---	tr	0.2	---	---	---	---	---
1293	1290	Dihydroedulan IIA	---	---	---	1.1	---	---	---	---	---	---	2.6	0.8	0.1
1295	1298	Carvacrol	---	---	---	---	0.3	---	---	---	---	---	---	---	---
1295	1293	Methyl myrtenate	---	---	---	0.1	---	---	---	---	---	---	---	---	---
1307	1295	*trans*-Sabinyl acetate	---	---	---	---	---	---	---	---	---	---	---	---	---
1330	1324	Methyl geranate	---	---	---	---	---	---	---	---	---	---	---	---	---
1330	1332	Bicycloelemene	---	---	---	0.2	---	---	1.1	0.5	---	---	---	---	---
1330	1335	δ-Elemene	0.2	1.1	---	---	---	---	0.9	0.4	---	0.2	1.0	1.4	0.5
1333	1325	Myrtenyl acetate	---	---	---	---	---	---	---	---	---	---	---	---	---
1342	1349	α-Cubebene	---	---	---	---	---	---	0.7	0.4	---	---	---	---	---
1345	1346	α-Terpinyl acetate	---	---	---	0.1	---	1.3	---	---	---	---	---	---	---
1364	1367	Cyclosativene	---	---	---	---	---	---	0.3	0.1	---	---	---	---	---
1364	1372	*iso*-Ledene	---	---	---	---	---	---	---	tr	---	---	---	---	---
1368	1373	α-Ylangene	---	---	---	tr	0.2	0.1	---	---	---	---	---	---	---
1374	1375	α-Copaene	---	---	---	0.1	3.1	0.7	3.2	1.5	---	0.2	---	0.3	0.2
1375	1380	Geranyl acetate	---	---	---	---	---	---	---	0.2	---	0.5	---	---	---
1377	1383	*cis*-β-Elemene	---	---	---	---	---	---	0.2	0.2	---	---	---	---	---
1377	1365	Eugenol	---	---	---	---	---	---	---	---	---	---	---	0.9	---
1382	1382	β-Bourbonene	---	---	---	tr	---	---	0.4	0.1	---	---	---	---	---
1386	1387	β-Cubebene	---	---	---	---	0.1	---	1.7	0.6	---	---	---	---	---
1387	1390	*trans*-β-Elemene	1.1	1.1	0.4	2.3	0.4	0.8	4.7	3.7	---	0.8	5.9	10.0	2.5
1400	1403	Methyl eugenol	---	---	---	0.1	---	---	---	---	---	---	---	---	---
1403	1405	Sesquithujene	---	---	---	---	---	---	---	---	---	---	---	---	---
1403	1408	(*Z*)-Caryophyllene	---	---	---	---	---	---	---	---	---	---	---	0.4	---
1405	1406	α-Gurjunene	---	---	---	0.1	---	---	0.1	0.1	---	0.3	---	---	---
1405	1415	β-Maaliene	---	---	---	---	---	---	0.1	---	---	---	---	---	---
1415	1416	*cis*-α-Bergamotene	---	0.4	---	0.2	---	---	0.1	0.1	---	---	0.2	0.3	---
1419	1417	(*E*)-Caryophyllene	13.9	52.6	1.9	24.3	1.6	4.9	6.4	6.3	1.7	11.3	12.2	14.4	2.3
1427	1432	γ-Elemene	---	---	---	---	---	0.3	0.7	---	---	---	---	---	---
1428	1424	2,5-Dimethoxy-*p*-cymene	0.1	---	---	---	---	---	---	---	---	---	---	---	---
1428	1430	β-Copaene	---	---	---	0.2	---	---	---	0.5	---	---	---	---	---
1428	1438	α-Maaliene	---	---	---	---	---	---	---	0.1	---	---	---	---	---
1431	1416	α-Santalene	---	---	0.1	---	---	---	---	---	---	---	---	---	---
1431	1432	*trans*-α-Bergamotene	0.2	0.1	---	tr	---	---	0.3	0.1	---	---	---	---	---
1433	1437	α-Guaiene	---	---	---	tr	---	0.2	---	---	---	---	---	---	---
1437	1438	Aromadendrene	0.2	0.2	---	0.1	---	---	0.6	0.5	---	---	0.2	0.4	0.4
1440	1440	(Z)-β-Farnesene	0.2	0.2	---	---	---	---	---	---	---	1.1	---	0.5	---
1444	1431	β-Gurjunene	---	0.1	---	---	---	---	---	---	---	---	---	0.9	0.2
1445	1445	Myltayl-4(12)-ene	---	---	---	0.1	---	---	---	---	---	---	---	---	---
1446	1453	Geranyl acetone	---	---	---	0.3	---	---	---	---	---	---	---	---	---
1447	1453	*trans*-Muurola-3,5-diene	---	---	---	---	0.1	0.4	0.2	0.1	---	---	---	---	---
1450	1452	(*E*)-β-Farnesene	---	---	0.3	0.2	---	0.1	0.3	0.2	---	---	---	---	---
1452	1457	Prezizaene	---	---	---	---	---	---	---	0.4	---	---	---	---	---
1454	1454	α-Humulene	2.1	5.9	0.3	3.2	1.1	4.8	6.4	8.8	0.2	6.9	1.1	1.6	0.5
1458	1457	*allo*-Aromadendrene	---	---	---	0.9	0.7	0.3	1.9	0.1	---	---	---	---	---
1460	1461	*cis*-Cadina-1(6),4-diene	---	---	---	---	0.1	0.1	---	---	---	---	---	---	---
1470	1472	*trans*-Cadina-1(6),4-diene	---	---	---	---	0.2	---	0.4	0.2	---	---	---	---	---
1471	1476	β-Chamigrene	---	---	---	0.1	---	---	---	---	---	---	0.6	2.6	0.7
1471	1475	γ-Gurjunene	---	---	---	---	---	---	---	---	---	---	---	1.5	---
1472	1482	γ-Curcumene	---	---	---	---	---	---	0.3	0.3	---	---	---	---	---
1473	1478	γ-Muurolene	0.2	---	---	0.1	0.3	0.1	0.3	0.3	---	0.4	---	---	---
1474	1476	Selina-4,11-diene	---	---	---	---	---	1.8	---	0.1	---	---	---	---	---
1477	1482	α-Amorphene	---	---	---	---	tr	0.2	---	0.1	---	---	---	---	---
1477	1479	*ar*-Curcumene	---	---	---	---	---	---	---	---	---	---	1.4	2.8	0.8
1477	1487	(*E*)-β-Ionone	---	---	---	0.1	---	---	---	---	---	---	---	---	---
1478	1483	*trans*-β-Bergamotene	---	---	---	---	---	---	0.2	---	---	---	---	---	---
1479	1464	9-*epi*-(*E*)-Caryophyllene	1.7	0.5	---	---	---	---	---	---	---	0.3	---	0.2	-
1480	1480	Germacrene D	0.6	0.4	---	3.6	tr	0.3	6.5	4.2	0.8	2.7	2.0	1.4	2.2
1481	1488	δ-Selinene	---	---	---	---	---	---	---	0.2	---	---	---	---	---
1482	1492	*cis*-β-Guaiene	---	---	---	---	---	---	0.3	---	---	---	---	---	---
1483	1487	Aristolochene	---	---	---	0.5	0.8	---	---	---	---	---	---	---	---
1488	1492	β-Selinene	0.6	0.3	0.3	0.8	5.4	2.4	1.4	1.8	---	0.3	---	2.0	0.6
1489	1496	Valencene	---	---	---	---	---	---	---	---	---	---	---	---	---
1490	1490	γ-Amorphene	---	---	---	0.1	0.1	0.4	1.3	0.8	---	---	0.8	1.6	---
1491	1493	α-Zingiberene	---	---	---	---	---	---	---	---	---	---	3.6	1.7	0.6
1494	1498	α-Selinene	---	---	---	---	1.4	2.2	---	---	---	---	1.0	---	---
1494	1497	Bicyclogermacrene	9.6	1.1	0.3	3.3	---	---	16.1	9.8	2.0	5.1	0.7	---	1.3
1496	1500	α-Muurolene	---	---	---	0.3	0.2	0.2	---	---	---	---	---	---	---
1496	1506	α-Bulnesene	---	---	---	---	---	---	0.5	0.3	---	---	---	---	---
1497	1502	*trans*-β-Guiaene	---	---	---	---	---	---	0.1	---	---	---	---	---	---
1500	1500	*n*-Pentadecane	---	---	---	---	---	---	---	---	---	---	---	---	---
1501	1505	(*E*,*E*)-α-Farnesene	1.6	0.7	0.4	---	0.4	0.3	---	---	---	0.7	---	---	---
1501	1495	γ-Amorphene	---	0.2	---	---	---	---	---	---	---	---	---	---	---
1502	1496	Viridiflorene	---	---	---	---	---	---	---	---	---	---	---	---	0.4
1503	1509	β-Curcumene	---	---	---	---	---	---	0.1	0.1	---	---	---	---	---
1506	1508	β-Bisabolene	---	---	---	0.1	0.1	0.6	0.7	0.3	---	---	1.4	2.0	0.4
1511	1512	γ-Cadinene	0.1	---	---	0.2	1.7	3.1	0.2	0.2	---	0.2	---	0.3	0.2
1511	1511	δ-Amorphene	---	---	---	---	---	---	---	---	---	---	---	---	---
1513	1519	Cubebol	---	---	---	---	0.1	---	0.4	0.2	---	---	---	---	---
1516	1518	δ-Cadinene	0.6	0.2	---	0.4	1.0	1.7	2.9	2.4	---	0.5	---	0.9	1.4
1517	1520	7-*epi*-α-Selinene	---	---	---	---	tr	1.1	---	---	---	---	1.0	1.1	---
1517	1521	Zonarene	---	---	---	---	---	---	0.1	0.3	---	---	---	---	---
1519	1521	*trans*-Calamenene	---	---	---	---	0.1	---	---	---	---	---	---	---	---
1520	1528	(*E*)-γ-Bisabolene	---	---	---	---	---	---	0.5	0.2	---	---	---	---	---
1523	1521	Eugenyl acetate	---	---	---	---	---	---	---	---	---	---	---	1.0	0.6
1524	1521	β-Sesquiphellandrene	---	---	---	---	---	---	---	---	---	---	0.7	0.5	---
1529	1528	*cis*-Calamene	---	---	---	---	---	---	---	---	---	---	---	0.3	---
1530	1536	*trans*-Cadina-1,4-diene	---	---	---	---	0.1	---	0.3	0.2	---	---	---	---	---
1535	1537	α-Cadinene	---	---	---	---	tr	0.2	---	---	---	---	---	---	---
1539	1544	α-Calacorene	---	---	---	---	0.5	0.4	---	---	---	---	---	---	---
1546	1549	α-Elemol	---	0.2	---	---	0.1	0.4	0.8	0.9	---	0.2	---	0.6	---
1557	1559	Germacrene B	---	---	---	---	---	0.7	0.6	0.2	---	---	0.3	---	0.2
1558	1561	(*E*)-Nerolidol	1.1	1.2	0.2	0.2	1.1	---	---	---	0.3	3.3	0.8	3.7	0.9
1560	1564	β-Calacorene	---	---	---	---	0.2	0.1	---	---	---	---	---	---	---
1564	1568	Palustrol	---	---	---	---	---	---	tr	0.1	---	---	---	---	---
1568	1571	Maaliol	---	---	---	---	---	0.2	tr	0.3	---	---	---	---	---
1570	1573	(3*E*,7*E*)-4,8,12-Trimethyltrideca-1,3,7,11-tetraene	---	---	---	0.1	---	---	---	---	---	---	---	---	---
1575	1576	Spathulenol	0.9	0.7	0.1	2.1	tr	0.2	1.2	0.9	0.2	1.3	---	1.2	0.2
1575	1582	Neryl isovalerate	---	---	---	---	---	0.2	---	---	---	---	---	---	---
1575	1574	Germacrene D-4-ol	---	---	---	---	---	---	---	---	---	---	---	---	---
1582	1577	Caryophyllene oxide	1.3	4.4	0.4	3.6	8.0	2.4	tr	0.3	0.2	4.1	2.0	4.5	1.6
1582	1582	*ar*-Turmerol	---	---	---	---	---	---	---	---	---	---	---	---	---
1583	1570	Dendrolasin	0.2	---	---	---	---	---	---	---	---	---	---	---	---
1583	1585	*epi*-Globulol	---	---	---	0.8	tr	0.9	---	---	---	---	---	---	---
1587	1590	Globulol	---	---	---	---	tr	0.4	1.0	1.6	---	---	---	---	---
1590	1581	Clovenol	---	---	---	---	---	---	---	---	---	---	---	0.2	---
1592	1594	Salvial-4(14)en-1-one	---	---	---	0.1	---	---	---	---	---	---	---	---	---
1593	1592	Viridiflorol	---	---	---	0.2	---	0.5	0.7	1.2	---	---	---	---	---
1593	1593	Scapanol ^g^	---	---	---	---	---	---	---	---	---	---	---	---	---
1594	1600	Guaiol	0.4	0.6	0.1	---	0.2	0.5	---	---	---	0.2	---	0.7	---
1594	1601	*trans*-β-Elemenone	---	---	---	---	tr	0.4	---	---	---	---	---	---	---
1596	1599	Cubeban-11-ol	---	---	---	0.1	---	---	tr	0.3	---	---	---	---	---
1599	1592	Humulene epoxide I	---	---	---	---	---	---	---	---	---	0.4	---	0.9	---
1600	1609	Rosifoliol	---	---	---	---	---	---	0.2	0.4	---	---	---	---	---
1604	1607	5,7-di-*epi*-Eudesmol	---	---	---	---	---	0.2	---	---	---	---	---	---	---
1604	1602	Ledol	---	---	---	0.3	---	---	---	---	---	0.2	---	---	---
1613	1618	1,10-di-*epi*-Cubenol	---	---	---	---	0.2	0.7	---	---	---	---	---	---	---
1616	1613	Humulene epoxide II	0.2	0.8	---	0.4	1.8	1.3	tr	0.2	---	2.8	---	0.6	0.6
1616	1611	Zingiberenol	---	---	---	---	---	---	---	---	---	---	---	0.3	---
1618	1623	Humulane-1,6-dien-3-ol	---	---	---	---	---	---	8.2	9.9	---	---	---	---	---
1620	1622	10-*epi*-γ-Eudesmol	---	---	---	---	tr	0.4	---	---	---	2.9	---	---	---
1625	1631	1-*epi*-Cubenol	0.2	0.3	---	---	0.2	0.3	---	1.3	---	---	---	---	---
1627	1618	*epi*-Cedrol	---	---	0.2	---	---	---	---	---	---	---	---	---	---
1628	1622	Alismol	---	---	---	---	---	---	---	---	---	---	---	---	0.4
1630	1630	γ-Eudesmol	---	---	---	---	0.3	1.7	0.5	0.7	---	---	---	---	---
1632	1631	Caryophylla-4(12),8(13)-dien-5α-ol	---	---	---	0.7	0.1	---	---	---	---	---	---	---	---
1634	1632	α-Acorenol	---	---	---	---	---	---	---	---	---	---	---	---	---
1636	1639	Caryophylla-4(12),8(13)-dien-5β-ol	---	---	---	0.9	0.3	0.4	---	---	---	---	---	---	---
1640	1638	τ-Cadinol	0.9	2.2	---	0.1	---	0.3	---	---	---	---	---	1.7	0.6
1640	1643	Cubenol	---	---	---	---	0.1	---	0.6	0.7	---	---	---	---	---
1642	1644	τ-Murrolol	---	---	---	0.2	0.1	0.4	---	0.4	---	0.2	---	0.6	---
1645	1644	α-Muurolol (= δ-Cadinol)	0.2	---	---	0.1	tr	0.1	0.2	0.7	---	---	---	---	---
1651	1649	β-Eudesmol	---	---	---	---	---	---	---	---	---	---	---	---	---
1652	1652	α-Cadinol	0.5	0.5	---	0.7	tr	1.3	1.0	1.7	---	0.3	0.2	2.0	0.5
1652	1647	1,2-Diacetoxy-4-allylbenzene	0.1	1.9	---	---	---	---	---	---	---	---	---	---	---
1653	1652	α-Eudesmol	---	---	---	---	0.6	1.1	---	---	---	---	---	1.0	---
1657	1658	Selin-11-en-4α-ol	---	---	---	0.2	tr	0.5	---	---	---	---	---	---	---
1662	1658	*neo*-Intermedeol	0.7	1.2	---	---	0.2	4.4	0.2	0.6	---	0.2	0.4	2.6	---
1668	1668	14-Hydroxy-9-*epi*-(*E*)-caryophyllene	---	0.6	---	0.6	0.3	0.5	---	---	---	0.4	0.2	1.0	---
1670	1675	Cadalene	---	---	---	---	0.1	---	---	---	---	---	---	---	---
1671	1676	Mustakone	---	---	---	---	0.1	---	---	---	---	---	---	---	---
1679	1681	γ-Bicyclofarnesal	---	0.9	0.6	---	---	---	---	---	---	1.2	---	0.6	0.4
1681	1685	α-Bisabolol	---	---	---	---	---	---	---	---	---	---	---	---	---
1684	1685	Germacra-4(15),5,10(14)-trien-1α-ol	---	---	---	0.1	---	---	---	---	---	---	---	---	---
1684	1683	*epi*-α-Bisabolol	---	---	---	---	---	---	---	---	---	---	---	---	---
1690	1693	Germacrone	---	---	---	---	0.2	1.6	---	---	---	---	---	---	---
1691	1694	Germacra-4(15),5,10(14)-trien-1β-ol	---	---	---	0.1	---	---	---	---	---	---	---	---	---
1696	1689	6α-Hydroxygermacra-1(10),4-diene	---	---	---	---	---	---	---	---	---	---	---	---	---
1701	1698	(2*Z*,6*Z*)-Farnesol	---	---	---	---	---	---	---	---	---	---	---	---	---
1702	1690	(Z)-*trans*-α-Bergamotol	---	0.3	---	---	---	---	---	---	---	---	---	---	---
1707	1714	Nootkatol	---	---	---	0.1	---	---	---	---	---	---	---	---	---
1713	1713	Pentadecanal	---	---	---	0.1	---	---	---	---	---	0.3	---	---	0.9
1738	1740	Mint sulfide	---	---	---	0.1	---	---	---	---	0.2	0.9	0.6	2.6	---
1739	1732	Zerumbone	---	---	---	---	---	---	---	---	0.2	3.9	---	---	---
1761	1759	Benzyl benzoate	---	---	---	---	---	---	2.5	3.0	---	0.3	---	0.3	---
1774	1760	(Z)-Lanceol	---	---	---	---	---	---	---	---	---	---	---	---	---
1799	1809	Ambrial	---	---	---	0.2	tr	0.5	---	---	---	---	0.4	2.8	2.4
1838	1838	Phytone	---	---	---	0.7	---	---	---	---	---	---	0.2	0.2	---
1848	1845	6,10,14-Trimethylpentadecan-2-one	---	---	---	---	---	---	---	---	---	---	---	---	---
1862	1864	Benzyl salicylate	---	---	---	---	---	---	0.5	0.8	---	---	---	---	---
2002	---	(*E*)-15,16-Bisnorlabda-8(17),11-dien-13-one	---	0.8	---	---	---	---	---	---	---	---	---	0.3	---
2104	2105	(*E*)-Phytol	---	0.2	---	0.9	---	---	---	---	---	0.2	---	0.6	---
		Monoterpene hydrocarbons	50.3	0.5	84.8	39.4	29.6	23.9	16.0	23.4	94.2	18.3	52.7	3.6	61.8
		Oxygenated monoterpenoids	3.8	12.0	1.7	2.0	34.8	25.3	1.1	2.9	0.1	16.2	0.4	0.0	0.8
		Sesquiterpene hydrocarbons	32.9	65.1	4.0	41.4	20.0	28.5	63.3	46.6	4.7	31.0	34.1	49.1	15.4
		Oxygenated sesquiterpenoids	6.6	13.9	1.6	11.6	14.0	21.3	14.8	22.2	0.9	21.6	3.6	22.2	5.2
		Others	0.2	2.9	0.0	4.0	0.8	0.5	3.1	4.1	0.2	2.3	3.8	9.5	4.0
		Total identified	93.8	94.4	92.1	98.4	99.2	99.5	98.3	99.2	100.0	89.4	94.6	84.4	87.2

RI_calc_ = Retention Indices determined with respect to a homologous series of *n*-alkanes on an HP-5ms column; RI_db_ = Retention Indices from the databases (NIST 17 and Wiley version 10); tr = trace (< 0.05%).

**Table 3:** Comparison of the major components of *Zingiber ottensii* rhizome essential oils from different geographical locations.

Major Components	Collection Location					
	Johor, Malaysia [37]	Petchaburi, Thailand [39]	Sabah, Malaysia [38]	Chiang Mai, Thailand [40]	Bandung, Indonesia [41]	Pù Hoạt Nature Reserve, Vietnam (This Work)
Sabinene	7.2	6.5	4.1	21.1	8.6	21.6
β-Pinene	5.1	4.3	1.3	6.8	3.5	11.7
*p*-Cymene	0.0	6.9	0.0	0.0	0.0	2.1
1,8-Cineole	3.3	1.2	0.6	3.9	5.8	4.3
γ-Terpinene	5.1	0.2	0.4	4.7	4.8	5.5
Terpinen-4-ol	16.8	11.2	3.2	19.7	16.6	17.1
α-Humulene	10.9	5.6	18.3	15.6	3.3	3.8
Zerumbone	25.6	40.1	36.7	0.0	14.2	12.5

**Table 4:** Major components in the leaf, stem, and rhizome essential oils of *Zingiber vuquangensis*.

Major Components	Vu Quang National Park [46]			Pù Hoạt Nature Reserve		
	Leaf	Stem	Rhizome	Leaf	Stem	Rhizome
α-Pinene	6.9	5.2	2.2	11.3	0.5	9.8
β-Pinene	24.7	26.1	8.0	38.5	3.1	45.0
Bornyl acetate	2.5	0.0	20.9	0.0	0.0	0.0
*trans*-β-Elemene	0.8	0.5	0.6	5.9	10.0	2.5
(*E*)-Caryophyllene	12.3	13.9	4.5	12.2	14.4	2.3
α-Humulene	7.7	8.2	9.6	1.1	1.6	0.5
Zerumbone	3.0	3.6	14.1	0.0	0.0	0.0

**Table 5:** Larvicidal activities of *Zingiber* essential oils from north-central Vietnam.

*Zingiber* Essential Oil	LC_50_ (95% Confidence Levels)	LC_90_ (95% Confidence Levels)	χ^2^	*p*
	Twenty-four-hour			
	*Aedes aegypti*			
*Z. cornubracteatum* (BE) rhizome	16.97 (15.49–18.42)	24.56 (22.65–27.31)	1.7 × 10^−6^	1.000
*Z. neotruncatum* rhizome	34.95 (32.49–37.37)	51.49 (48.32–55.56)	6.625	0.036
*Z. nudicarpum* (PH) leaf	19.30 (17.95–20.56)	23.74 (22.40–25.40)	0.0	1.000
*Z. nudicarpum* (PH) rhizome	23.44 (21.94–25.17)	31.81 (29.07–36.31)	1.1 × 10^−3^	0.999
*Z. ottensii* rhizome	38.16 (35.50–40.89)	57.87 (53.60–63.62)	3.286	0.193
*Z. recurvatum* rhizome	20.90 (18.45–23.30)	36.35 (32.79–41.63)	3.697	0.157
Permethrin (control)	0.0094 (0.0082–0.0107)	0.0211 (0.0185–0.0249)	57.6	0.000
	*Aedes albopictus*			
*Z. cornubracteatum* (BE) rhizome	12.72 (10.43–14.41)	21.56 (19.43–25.10)	4.6 × 10^−6^	1.000
*Z. neotruncatum* rhizome	21.50 (19.71–23.52)	31.99 (28.97–36.94)	0.0402	0.980
*Z. nudicarpum* (PH) leaf	22.33 (20.93–23.86)	31.12 (27.52–36.51)	0.2454	0.970
*Z. nudicarpum* (PH) rhizome	28.05 (25.80–30.41)	42.09 (38.92–46.29)	3.205	0.201
*Z. ottensii* rhizome	19.79 (17.91–21.75)	30.81 (27.81–35.76)	0.03531	0.983
*Z. recurvatum* rhizome	45.58 (43.24–47.49)	58.58 (55.18–63.22)	0.01253	0.994
Permethrin (control)	0.0024 (0.0021–0.0026)	0.0042 (0.0038–0.0049)	4.64	0.031
	*Culex quinquefasciatus*			
*Z. cornubracteatum* (BE) rhizome	24.31 (21.79–26.84)	41.34 (37.72–46.34)	25.99	0.000
*Z. neotruncatum* rhizome	33.58 (31.39–36.00)	42.76 (39.94–46.48)	6.675	0.036
*Z. nudicarpum* (PH) leaf	12.44 (8.78–15.67)	44.29 (39.05–51.64)	34.20	0.000
*Z. nudicarpum* (PH) rhizome	11.50 (9.65–13.36)	27.15 (23.92–31.87)	30.33	0.000
*Z. ottensii* rhizome	27.19 (25.04–29.58)	40.09 (36.82–44.64)	8.377	0.015
*Z. recurvatum* rhizome	31.67 (29.30–34.05)	47.02 (43.94–50.94)	1.300	0.522
Permethrin (control)	0.0188 (0.0173–0.0206)	0.0294 (0.0270–0.0326)	24.1	0.000
	Forty-eight-hour			
	*Aedes aegypti*			
*Z. cornubracteatum* (BE) rhizome	16.32 (14.72–17.84)	24.52 (22.50–27.54)	9.8 × 10^−6^	1.000
*Z. neotruncatum* rhizome	33.91 (31.45–36.33)	50.42 (47.26–54.46)	8.156	0.017
*Z. nudicarpum* (PH) leaf	16.97 (15.76–18.32)	22.22 (20.59–24.53)	0	1.000
*Z. nudicarpum* (PH) rhizome	22.73 (21.09–24.63)	31.31 (28.88–35.35)	8.8 × 10^−3^	0.996
*Z. ottensii* rhizome	36.59 (33.77–39.48)	57.61 (53.69–62.84)	7.113	0.029
*Z. recurvatum* rhizome	17.16 (14.00–19.79)	34.57 (30.87–40.26)	3.687	0.158
	*Aedes albopictus*			
*Z. cornubracteatum* (BE) rhizome	12.32 (10.36–13.78)	19.57 (17.61–23.23)	0	1.000
*Z. neotruncatum* rhizome	19.97 (18.07–21.98)	31.24 (28.16–36.33)	0.0528	0.974
*Z. nudicarpum* (PH) leaf	14.31 (12.03–16.52)	29.76 (27.71–32.79)	2.548	0.467
*Z. nudicarpum* (PH) rhizome	23.80 (21.40–26.25)	39.59 (36.06–44.53)	12.33	0.002
*Z. ottensii* rhizome	17.30 (15.15–19.24)	28.94 (25.97–33.94)	0.02453	0.988
*Z. recurvatum* rhizome	42.47 (40.04–44.88)	57.289 (54.63–61.53)	1.031	0.597
	*Culex quinquefasciatus*			
*Z. cornubracteatum* (BE) rhizome	11.48 (5.78–15.28)	33.79 (29.71–40.16)	9.085	0.011
*Z. neotruncatum* rhizome	29.91 (27.77–32.33)	41.42 (38.31–45.66)	13.46	0.001
*Z. nudicarpum* (PH) leaf	4.901 (3.982–5.817)	18.31 (14.98–23.84)	83.59	0.000
*Z. nudicarpum* (PH) rhizome	6.687 (4.942–8.197)	18.85 (16.52–22.30)	49.77	0.000
*Z. ottensii* rhizome	24.64 (22.58–26.98)	36.94 (33.58–41.98)	1.512	0.470
*Z. recurvatum* rhizome	7.965 (4.368–10.991)	33.52 (26.87–46.73)	2.905	0.234

LC_50_ = medial lethal concentration, LC_90_ = 90% lethal concentration, χ^2^ and *p* = goodness-of-fit chi-square value and *p*-value, BE = Bến En National Park, PH = Pù Hoạt Nature Reserve.

**Table 6:** Antibacterial and antifungal activities of *Zingiber* essential oils from north-central Vietnam.

*Zingiber* Essential Oil (EO)	Gram-Positive Bacteria			Gram-Negative Bacteria			Yeast
	*Enterococcus faecalis* ATCC 299212	*Staphylococcus aureus* ATCC 25923	*Bacillus cereus* ATCC 14579	*Escherichia coli* ATCC 25922	*Pseudomonas aeruginosa* ATCC 27853	*Salmonella enterica* ATCC 13076	*Candida albicans* ATCC 10231
	MIC (μg/mL)						
*Z. cornubracteatum* (BE) leaf EO	128	128	na	64	na	na	na
*Z. cornubracteatum* (BE) rhizome EO	32	32	64	256	na	na	256
*Z. cornubracteatum* (BE) stem EO	128	128	na	64	na	na	na
*Z. cornubracteatum* (PM) leaf EO	64	64	128	128	na	na	16
*Z. cornubracteatum* (PM) rhizome EO	32	64	128	64	256	na	16
*Z. nudicarpum* (PH) leaf EO	32	16	16	na	128	na	16
*Z. nudicarpum* (PH) rhizome EO	2	8	1	64	16	na	16
*Z. ottensii* leaf EO	64	64	64	na	16	na	64
*Z. ottensii* rhizome EO	8	64	32	na	na	na	8
*Z. recurvatum* leaf EO	16	64	128	na	na	na	128
*Z. recurvatum* rhizome EO	16	128	64	na	256	256	64
*Z. vuquangensis* leaf EO	16	16	16	na	na	na	32
Streptomycin	256	256	128	32	256	128	nt
Nistatin	nt	nt	nt	nt	nt	nt	8
Cyclohexamide	nt	nt	nt	nt	nt	nt	32
	IC_50_ (μg/mL)						
*Z. cornubracteatum* (BE) leaf EO	45.67	45.67	na	18.78	na	na	na
*Z. cornubracteatum* (BE) rhizome EO	10.45	9.34	18.79	68.99	na	na	99.34
*Z. cornubracteatum* (BE) stem EO	46.78	19.78	na	67.89	na	na	na
*Z. cornubracteatum* (PM) leaf EO	20.78	9.34	45.67	45.67	na	na	5.67
*Z. cornubracteatum* (PM) rhizome EO	9.56	13.56	43.67	20.45	100.34	na	7.89
*Z. nudicarpum* leaf EO	16.33	8.54	8.57	na	65.44	na	8.67
*Z. nudicarpum* rhizome EO	1.33	4.35	0.567	33.22	8.66	na	8.99
*Z. ottensii* leaf EO	32.66	32.33	33.77	na	7.99	na	32.33
*Z. ottensii* rhizome EO	2.57	17.89	15.56	na	na	na	3.56
*Z. recurvatum* leaf EO	3.99	13.67	55.89	na	na	na	67.74
*Z. recurvatum* rhizome EO	6.46	36.87	32.33	na	108.99	112.67	25.67
*Z. vuquangensis* leaf EO	15.66	8.56	7.33	na	na	na	16.33

BE = Bến En National Park, PM = Pù Mát National Park, MIC = minimum inhibitory concentration, IC_50_ = median inhibitory concentration, na = not active, nt = not tested.
